# Inertial Particle Migration in Contraction–Expansion Microchannels: Effects of Rectangular and Hook-Shaped Microstructures on Secondary Flow and Inertial Lift Competition

**DOI:** 10.3390/mi17101127

**Published:** 2026-09-27

**Authors:** Qing Liu, Hongchao Fang, Yongming Zhang, Huaichao Wang, Shu Han, Di Huang

**Affiliations:** 1 Air Force Logistics Academy, Xuzhou 221000, China; kqw_260806@163.com (Q.L.); al_ym@163.com (Y.Z.); wanghuaichao123@163.com (H.W.); 2 School of Mechanical and Electrical Engineering, China University of Mining and Technology, Xuzhou 221116, China; huangdi1105@cumt.edu.cn

**Keywords:** inertial microfluidics, hook-shaped microstructure, contraction-expansion channel, inertial migration, secondary flow

## Abstract

Inertial microfluidics offers significant advantages for bioparticle manipulation, including structural simplicity, label-free and external-field-free operation, and high throughput. In this study, numerical simulations and polydimethylsiloxane (PDMS)-based microfluidic experiments were combined to systematically investigate the effects of embedding depth (*H*) and expansion segment length (*L*) on particle inertial migration in contraction–expansion array (CEA) channels incorporating embedded rectangular and hook-shaped microstructures. The results demonstrate that particle equilibrium positions are governed by the competition among inertial lift, secondary flow drag, and the effective range over which the secondary flow acts. For both channel configurations, increasing *H* simultaneously enhances the peak secondary flow intensity and enlarges its effective range, thereby driving particles toward the sidewalls. In contrast, for the two microstructures studied here, the secondary flow intensity is only weakly sensitive to *L*. In rectangular microstructures, however, the secondary flow is already fully developed within short expansion cavities; further increasing *L* does not enlarge the effective range but instead weakens the modulation effect of the secondary flow on particles, allowing inertial lift to become more prominent. In hook-shaped microstructures, the secondary flow is not fully developed in short cavities, and increasing *L* promotes its development and markedly enlarges its effective range, thereby strengthening lateral particle regulation. It can be anticipated that, once the secondary flow is fully developed, its effective range will saturate, and further increasing *L* will weaken the modulation effect of the secondary flow on particles, allowing inertial lift to dominate again. Using the experimental structure, particle separation experiments were performed, achieving efficient label-free inertial separation of 7 μm and 15 µm particles, with a recovery rate of 99.3% for 15 μm particles and both purity and recovery exceeding 98%. This work elucidates the key role of the effective range of secondary flow in inertial particle migration and provides theoretical guidance for the rational design of CEA-based microfluidic devices.

## 1. Introduction

Microfluidic technology enables precise manipulation of fluids and particles within channels with characteristic dimensions ranging from tens to hundreds of micrometers, thereby significantly advancing biological and chemical analysis. Often referred to as “lab-on-a-chip,” this technology integrates key processes such as focusing [1], separation [2], and purification [3] onto miniaturized platforms. These devices offer distinct advantages, including minimal reagent consumption, rapid analysis, high throughput, and portability, making them highly attractive for applications in point-of-care diagnostics, environmental monitoring, and single-cell analysis.

Microfluidic techniques for particle manipulation can be categorized into active methods, passive methods, and hybrid actuation strategies that integrate both, collectively forming a continuous spectrum of microfluidic control solutions rather than two mutually exclusive categories [4]. Active methods rely on external force fields—including dielectrophoresis (DEP) [5], magnetic [6], acoustic [7], or optical [8] fields—to regulate particle motion, with core advantages of high manipulation precision, strong real-time tunability, and on-demand selective sorting and dynamic control. The performance characteristics and limitations vary significantly across different actuation mechanisms: for instance, cell separation based on tilted-angle standing surface acoustic waves enables label-free, non-contact, and high-efficiency sorting, with good biocompatibility verified by cell viability and integrity assays in previous studies [9]. However, some active manipulation systems still require bulky driving and signal generation devices with relatively high energy consumption. Notably, the risk of biological sample damage is closely related to parameters such as actuation frequency, input power, and exposure duration, rather than being an inherent property of all active methods.

In contrast, passive methods manipulate particles without external field input, relying solely on hydrodynamic effects and channel geometry. Representative passive techniques include inertial focusing [10], deterministic lateral displacement (DLD) [11], viscoelastic focusing [12], and hydrodynamic filtration [13]. These approaches have prominent advantages in high-throughput continuous processing scenarios due to their simple structure, label-free operation, ease of fabrication, and low operational cost. Nevertheless, they suffer from limited regulatory flexibility, with performance highly dependent on channel geometric design, making it generally difficult to achieve dynamically adjustable selective manipulation. In terms of biocompatibility, passive manipulation has low potential for biological damage due to the absence of external field intervention; however, the shear stress level and flow field distribution within the channel may still affect the viability and function of biological particles.

To combine the technical advantages of both approaches, active-passive hybrid manipulation strategies have emerged as an important development direction. Integrating acoustic actuation with passive microvalve structures represents a typical technical route in this field, which enables dynamic intra-droplet selection and overcomes the limitation that fixed passive channels cannot be tuned on demand. Intra-droplet particle switching based on bulk acoustic waves, combined with geometric design of passive droplet splitting, allows precise acoustic routing of encapsulated particles [4,14]. Such hybrid schemes leverage the hydrodynamic fundamentals of passive structures while retaining the flexibility of active regulation, providing more abundant design ideas for complex biological sample processing. For the present study, which focuses on the mechanism of inertial particle manipulation in contraction–expansion array channels, the passive inertial microfluidic scheme is more suitable for systematic mechanistic investigation, as it introduces no external field interference and allows independent analysis of the intrinsic correlation between geometric parameters and hydrodynamic effects. Meanwhile, the geometric regulation laws obtained in this study can also provide theoretical support for the optimal design of passive functional units in hybrid manipulation systems.

Among passive techniques, inertial microfluidics has emerged as a prominent area of research owing to its ability to achieve high-throughput, sheath-free particle focusing and separation by exploiting fluid inertia at moderate Reynolds numbers. Inertial focusing is governed by the interplay between two primary forces: the inertial lift force ($F_{L}$), which arises from the non-uniform velocity profile, and the drag force induced by secondary flows ($F_{D}$). Notably, secondary flow is a general category of transverse flows, encompassing various specific types, including classical Dean flow, which is the specific type induced by centrifugal effects in curved channels. In straight channels, particles migrate to specific equilibrium positions solely under the influence of inertial lift forces. However, the governing force balance varies depending on the channel geometry: in curved channels, the competition between the inertial lift force and the Dean drag force determines the final equilibrium positions of particles; whereas in straight channels incorporating contraction–expansion (CEA) structures, the competition between the inertial lift force and the geometry-induced secondary flow drag force determines the final equilibrium positions. This distinction enables precise, size-based particle manipulation in a sheath-free manner across both configurations.

Extensive research has been conducted to optimize particle manipulation across various channel configurations. In terms of channel geometry, Liu et al. [15] systematically investigated the equilibrium positions of particles in contraction–expansion array (CEA) channels, demonstrating that focusing patterns strongly depend on the aspect ratio and expansion angle. Zhou et al. [16] further revealed that modulating the channel aspect ratio alone enables complete separation of particles and rare cells with a recovery rate exceeding 99%. To regulate the secondary flow, Wang et al. [1] introduced multiple sheath streams into a contraction–expansion microchannel, which strengthened the Dean vortices and improved focusing and sorting performances by 31.9% and 55.2%, respectively, when the sheath streams increased from one to two. Yan et al. [17] combined an expansion-contraction array with slanted grooves in a double-layer channel, extending the operational flow rate range for particle focusing with a focusing efficiency of over 95%. Shamloo et al. [18] found that coupling CEA structures with curved geometries can achieve effective separation within channel lengths reduced by 4.34 mm.

For biological applications, Yeh et al. [19] developed an optimized CEA device for the label-free isolation of natural killer cells, achieving a recovery rate of 70.9% and a purity of 91.2%. Zhou et al. [20] demonstrated high-throughput sheathless separation of flexible microalgae in spiral-coupled contraction-expansion channels, achieving purities of 100% for Chlorella vulgaris and 76.5% for *Haematococcus pluvialis*. Song et al. [21] established design rules for size-based cell sorting and sheathless cell focusing by hydrophoresis, identifying channel width as a key geometric parameter, while noting that the oblique angle of the slanted grooves has no significant effect on performance.

Despite these advances, a systematic understanding of how the geometric dimensions and morphology of microstructures in contraction–expansion array (CEA) channels govern inertial particle manipulation remains limited. In particular, the detailed mechanisms by which key geometric parameters—such as embedding depth and expansion segment length—modulate the competition between inertial lift and secondary flow have not been fully elucidated.

Secondary flow has been widely recognized as a key factor governing particle manipulation in contraction–expansion array (CEA) channels. However, current understanding primarily focuses on the influence of secondary flow intensity on particle migration behavior. Previously, Huang et al. [22] experimentally validated the potential of hook-shaped microstructures for particle trapping, focusing, and separation, and reported that the secondary flow intensity increases with the expansion segment length (*L*), embedding depth (*H*), and main channel width (*W*). However, these trends were mainly inferred from experimental observations. In particular, the underlying mechanism for the anomalous phenomenon where the secondary flow intensity increases with L has not been sufficiently explained.

To address this gap, this study combines numerical simulations with systematic experiments on both rectangular and hook-shaped channels. Based on the characterization of particle migration in straight channels, we comparatively analyzed the regulatory mechanisms of *L* and *H* on particles in rectangular and hook-shaped microstructures. Through comparative verification of numerical simulations and experiments, a key physical mechanism was revealed: in addition to the local vortex intensity, the spatial extension capability of the secondary flow vortex along the flow direction and its effective acting distance exert a decisive influence on the inertial migration and focusing of particles. When the expansion cavity length increases, if the vortex can fully extend within the cavity, it prolongs the path of lateral perturbation acting on particles, thereby enhancing the regulatory effect of the secondary flow; conversely, if the vortex reaches a saturated spatial scale and cannot extend further with the cavity, the over-extended expansion region forms a quiescent zone devoid of secondary flow regulation, resulting in a weakened regulatory effect of the secondary flow on particles.

Through the systematic integration of numerical simulations and experiments, this study elucidates the interplay between vortex size and the competition between inertial lift and secondary flow drag, providing a theoretical foundation for the rational design of inertial microfluidic devices.

## 2. Theoretical Background

### 2.1. Inertial Lift Force

Owing to the extremely small characteristic dimensions and low flow velocities in microfluidic devices, microfluidic flows have traditionally been regarded as operating in the low-Reynolds-number regime. The Reynolds number (Re) is a dimensionless parameter that quantifies the ratio of inertial forces to viscous forces and is defined as follows [23]:

(1) $$Re=\frac{\rho U_{f}D_{h}}{\mu }$$

where ρ is the fluid density, $U_{f}$ is the average flow velocity, μ is the dynamic viscosity, and $D_{h}$ is the hydraulic diameter. For a rectangular cross-section channel, the hydraulic diameter is expressed as $D_{h}=2wh/(w+h)$, where w and h denote the width and height of the rectangular cross-section, respectively.

In inertial microfluidics, the channel Reynolds number typically ranges between the Stokes regime and the turbulent regime. Under these conditions, the flow remains laminar, yet inertial effects become significant. Inertial migration was first reported by Segré and Silberberg [24] in 1962: at finite Reynolds numbers, suspended particles in a circular pipe migrate to a radial position approximately 0.6 times the tube radius from the center, forming an annular focusing band. In rectangular channels, particles focus toward the central regions of the long walls, whereas in square channels, particles focus at the centers of the four channel faces. This phenomenon originates from the Poiseuille velocity profile in straight channels, where the velocity gradient exerts a shear-induced inertial lift force that drives particles toward the wall. As particles approach the wall, the symmetric wake generated by particle rotation is disrupted by the wall, producing a wall-induced inertial lift force that repels particles away from the wall [25]. The balance between these two forces determines the equilibrium positions of particles, and their resultant is defined as the inertial lift force. Asmolov [26] derived the expression for the inertial lift force $F_{L}$ as:

(2) $$F_{L}=\frac{C_{L}\rho U_{m}^{2}a_{p}^{4}}{D_{h}^{2}}$$

where $C_{L}$ is the dimensionless lift coefficient, $U_{m}$ is the maximum flow velocity, and $a_{p}$ is the particle diameter.

### 2.2. Secondary Flow

Secondary flow refers to the general category of transverse flows induced by channel geometry. In curved channels, the combined effects of the radial pressure gradient and centrifugal force drive the fluid in the central region outward. Simultaneously, owing to the enclosed channel geometry, the fluid near the outer wall is compressed and recirculates along the top and bottom walls, thereby forming two symmetrical counter-rotating vortices across the channel cross-section. This specific type of secondary flow is known as Dean flow [27]. In curved channels, inertial migration of particles is governed by the synergistic interplay between the inertial lift force and the Dean drag force. The Dean drag force is typically approximated by Stokes drag and expressed as:

(3) $$F_{D}=3\pi \mu a_{p}U_{D}$$

where μ is the dynamic viscosity of the fluid, $a_{p}$ is the particle diameter, and $U_{D}$ is the Dean velocity. Ookawara et al. [28] derived an empirical correlation for the average Dean velocity through numerical simulations as $U_{D}=1.8\times 10^{-4}{De}^{1.63}$.

Previous studies have demonstrated that embedding contraction–expansion (CEA) structures within straight channels also induces secondary flows, which arise from the abrupt changes in the cross-sectional area rather than the centrifugal effects in curved channels [29]. In such configurations, the competitive interplay between the geometry-induced secondary flow drag and the inertial lift force determines the final equilibrium positions of particles. Because the net forces acting on particles of different sizes differ, their corresponding equilibrium positions vary accordingly, establishing the physical basis for size-based inertial focusing and separation.

## 3. Materials and Methods

### 3.1. Device Design and Fabrication

In this study, two microchannel configurations incorporating rectangular and hook-shaped microstructures, respectively, were designed. Each channel consists of two primary sections: (i) a straight rectangular main channel with a low aspect ratio, serving as the primary region for inertial particle migration, and (ii) arrays of microstructures symmetrically and periodically arranged along both sidewalls of the main channel. The main straight channel for the rectangular microstructures has a fixed width (*W*) of 120 μm and a uniform height of 60 μm, whereas the main straight channel for the hook-shaped microstructures has a fixed width (*W*) of 180 μm and the same height of 60 μm. The embedding depth of both the rectangular and hook-shaped microstructures is denoted as *H*, and the expansion segment length is denoted as *L*. In addition, the distance from the inlet to the first microstructure is 5 mm, and the length of the outlet section after the microstructures is 1.5 mm.

A total of 40 symmetric microstructure pairs are incorporated along the channel, forming a contraction–expansion array (CEA). All experimental images were captured at the exit of the 40th pair of microstructures (i.e., at the channel outlet). Notably, each individual hook-shaped microstructure is formed by tangentially joining a quarter-circular arc of radius $R_{1}$ with two semi-circular arcs of radius $R_{2}$, satisfying the geometric relation $R_{1}=4R_{2}$ (Figure 1).

The expansion segment width remains equal to the main channel width W. For the rectangular microstructures, the pitch of a single microstructure pair is fixed at $p_{\mathrm{rect}}=60\;\mu m$. For the hook-shaped microstructures, the pitch is equal to $R_{1}$, and the radii $R_{1}$ and $R_{2}$ depend on the embedding depth H, satisfying the geometric relation $R_{1}=4R_{2}$. Once H is specified, $R_{1}$ and $R_{2}$ can be determined from the tangential constraints of the hook-shaped geometry.

For the basic CEA channels used for studying inertial migration (without a branched outlet), the total channel length is calculated as $L_{\mathrm{total}}=L_{\mathrm{in}}+N\left(p+L\right)+L_{\mathrm{out}}$, where $L_{\mathrm{in}}=5\;\mathrm{mm}$ is the inlet development length, N=40 is the number of microstructure pairs, p is the pitch ($p_{\mathrm{rect}}=60\;\mu m$ for rectangular microstructures and $p_{\mathrm{hook}}=R_{1}$ for hook-shaped microstructures), L is the expansion segment length, and $L_{\mathrm{out}}=1.5\;\mathrm{mm}$ is the outlet section length. Since L is a variable in this study, $L_{\mathrm{total}}$ varies accordingly.

As shown in Figure 2, to enable size-based particle separation, the channel outlet was designed as a trifurcated structure. The main channel with a width of 180 μm expands to a total width of 540 μm at the outlet region and is then equally divided into three branches, each with a width of 180 μm, yielding a branch width ratio of 1:1:1. The center branch has a channel length of 2350 μm, while the two side branches are slightly longer due to their curved geometry. This outlet configuration was used only for the separation experiments reported in Section 4.4.

For the calculation of the hydraulic diameter $D_{h}$ and Reynolds number Re, the characteristic cross-section is taken as the main straight channel cross-section. For the channel embedded with rectangular microstructures, the width is W=120 μm and the height is 60 μm; for the channel embedded with hook-shaped microstructures, the width is W=180 μm and the height is 60 μm.

The microfluidic chip was fabricated using standard soft lithography. Briefly, a negative photoresist (SU-8 2050, MicroChem, Westborough, MA, USA) was spin-coated onto a silicon wafer, followed by ultraviolet (UV) exposure and development to produce a master mold bearing the microstructures. Subsequently, polydimethylsiloxane (PDMS) prepolymer and curing agent (Sylgard 184, Dow Corning, Midland, MI, USA) were thoroughly mixed at a mass ratio of 10:1, poured over the mold surface, degassed in a vacuum desiccator, and thermally cured at 80 °C for 2 h. After curing, the PDMS replica was peeled off, and inlet and outlet holes were punched using a biopsy puncher. The PDMS slab and a glass substrate were then treated with oxygen plasma and bonded together. The assembled device was finally heated in an oven at 120 °C for 3 h to ensure bonding strength and hermetic sealing.

### 3.2. Experimental Setup and Sample Preparation

#### 3.2.1. Sample Preparation

Polystyrene microsphere stock suspensions (mass concentration: 250 mg/mL; Tianjin Beisile Chromatography Technology Development Center, Tianjin, China) with diameters of 7 μm, 10 μm, and 15 μm were used as test particles. The particle density of polystyrene was taken as 1.05 g/cm^3^.

The number concentration of each stock suspension was calculated from the mass concentration and particle density. The total volume of microspheres per milliliter of stock solution was derived as the ratio of total microsphere mass to particle density. The single-particle volume was calculated as $v_{p}=\frac{\pi {d_{p}}^{3}}{6}$, where $d_{p}$ is the particle diameter. The calculated stock number concentrations are listed below:

- 7 μm particles: ~$1.33\times 10^{9}$ particles/mL
- 10 μm particles: $\sim 4.55\times 10^{8}$ particles/mL
- 15 μm particles: $\sim 1.35\times 10^{8}$ particles/mL

Working suspensions were prepared by serial dilution with deionized water, with a final mass fraction of 0.5 wt% Tween 20 (Sinopharm Chemical Reagent Co., Ltd., Shanghai, China). Surfactant molecules adsorb onto microsphere surfaces to form a steric hindrance layer, which effectively suppresses interparticle aggregation.

For particle migration experiments, the final particle number concentration was adjusted to $1\times 10^{5}\sim 2\times 10^{6}$ particles/mL. For particle separation experiments, 1 mL of mixed working suspension was prepared by diluting 7 μm and 15 μm stock suspensions to final concentrations of $1.5\times 10^{5}$ particles/mL and $5\times 10^{4}$ particles/mL, respectively. All working suspensions were vortexed for 3 min and sonicated at 40 kHz for 3 min immediately before injection to ensure uniform dispersion. The syringe was mounted vertically on the syringe pump to minimize particle sedimentation inside the syringe barrel. Absolute ethanol (purity ≥ 99.7%; Sinopharm Chemical Reagent Co., Ltd., Shanghai, China) and deionized water were used to flush the channels before each experiment.

All experiments were conducted at a controlled ambient temperature of 24±1 °C. Due to the very low dosage of Tween 20 (0.5 wt%), the physical properties of the working suspension are close to those of pure water. The working suspension had a density of $\rho =1001\;kg/m^{3}$ and a dynamic viscosity of $\mu =1.03\times 10^{-3}\;Pa\cdot s$.

#### 3.2.2. Experimental Setup and Imaging Protocol

The experimental setup consisted primarily of an inverted bright-field microscope (STL-MF52, Suzhou Furunruiyang Scientific Instrument Co., Ltd., Suzhou, China) equipped with a high-speed charge-coupled device (CCD) camera (STL-P2301uA, Suzhou Furunruiyang Scientific Instrument Co., Ltd., Suzhou, China), a precision syringe pump (LSP01-1A, Suzhou Wenhao Microfluidic Technology Co., Ltd., Suzhou, China), and an ultrasonic cleaner (KQ-100VDB, Kunshan Ultrasonic Instrument Co., Ltd., Kunshan, China). Microfluidic connections were made using polytetrafluoroethylene (PTFE) tubing and syringe needles (inner/outer diameter: 0.9 mm/1.2 mm).

A 20× objective lens with a numerical aperture (NA) of 0.45 was used for all observations, yielding a calibrated spatial resolution of 0.322 μm/pixel. The depth of field of the optical system was approximately 3–5 μm, and only particles within the focal plane were included in trajectory statistics. The observation position was fixed at the outlet of the 40th microstructure pair for all migration experiments. Before image acquisition, the suspension was pumped through the channel for at least 1 min to ensure fully developed and stable flow.

Bright-field videos of particle migration were acquired at a frame rate of 364 fps with an exposure time of 150 μs. Notably, motion blur occurred only along the flow direction (x-axis) and did not affect the measurement of particle lateral positions (y-axis). Quantitative statistics were based on the lateral center of each particle, so the exposure time setting did not compromise measurement accuracy.

Recorded video sequences were imported into ImageJ software (version 1.46r, National Institutes of Health, Bethesda, MD, USA) for analysis. To visualize particle trajectories, a standard deviation projection of 500 consecutive frames was performed using the “Z Project” function in ImageJ’s Stacks menu.

Each geometric parameter and flow rate condition was repeated in at least three independent experimental runs using separate PDMS chips and freshly prepared particle suspensions (n>3), independent replicates). Multiple fields of view within a single chip were treated as technical replicates. The 500 consecutive frames from a single video are temporally correlated and were not treated as independent observations. All quantitative results are presented as mean ± standard deviation (SD).

It should be noted that the present imaging setup is based on top-view bright-field microscopy, which resolves only the lateral (x–y) particle distribution. The vertical (z) coordinate and the exact three-dimensional equilibrium positions cannot be resolved. Therefore, all statements regarding particle focusing and migration in this work are restricted to the projected lateral distributions, and no claims are made about top/bottom-wall equilibrium or stable three-dimensional trapping. The 500 consecutive frames are a temporal stack rather than a spatial z-stack, and they are not treated as independent observations.

#### 3.2.3. Particle Counting and Separation Performance Quantification

Particle enumeration was performed using a standard improved Neubauer hemocytometer under bright-field microscopy.

- Inlet concentration calibration: Prior to injection, the homogenized inlet suspension was sampled and diluted to an appropriate counting concentration. Each sample was loaded into the hemocytometer counting chamber, and particles in the four large corner squares were counted. The final inlet number concentration was determined by averaging three independent sampling counts, and the total inlet particle number was calculated as the product of concentration and total injected suspension volume.
- Outlet collection and counting strategy: After each separation run, effluents from the three outlet branches were collected separately in pre-weighed centrifuge tubes. Consistent with the size-based separation performance of the device, effluents from the two side branches were pooled and fully homogenized for counting 7 μm particles, while effluent from the central branch was analyzed separately for 15 μm particles. Each outlet sample was counted in triplicate, and the mean count was used for subsequent calculations.
- Performance metrics and mass balance:

The recovery rate of target particles was defined as:

(4) $${\mathrm{Recovery}}_{\mathrm{target}}=\frac{N_{\mathrm{target,outlet}}}{N_{\mathrm{target,inlet}}}\times 100\%$$

where $N_{\mathrm{target,inlet}}$ is the total number of target particles introduced at the inlet, and $N_{\mathrm{target,outlet}}$ is the total number of target particles collected at the corresponding outlets (sum of two side outlets for 7 μm particles; central outlet only for 15 μm particles).

Purity is defined as the percentage of target particles in a given outlet relative to the total number of particles collected at that outlet:

(5) $$\mathrm{Purity}=\frac{N_{\mathrm{target},\mathrm{outlet}}}{N_{\mathrm{total},\mathrm{outlet}}}\times 100\%$$

where $N_{\mathrm{target},\mathrm{outlet}}$ is the number of target particles (7 μm or 15 μm) collected at the outlet, and $N_{\mathrm{total},\mathrm{outlet}}$ is the total number of particles collected at the same outlet. For the side outlets, the two outlets were combined for the calculation of 7 μm particle purity.

All separation performance results are presented as mean ± SD from three independent experimental runs.

### 3.3. Numerical Simulation

To elucidate the underlying physical mechanism of the secondary flow, three-dimensional fluid-only simulations were performed using the COMSOL Multiphysics 6.0 software. The physical field was set to laminar flow, and the steady-state incompressible Navier–Stokes (N–S) equations were solved with water as the working fluid. To accurately capture the complex vortex structures, a P2/P2 discretization scheme (second-order elements for both velocity and pressure) was adopted. It should be noted that the displayed simulation planes correspond to the Z-direction mid-plane at 30 μm, which is half of the 60 μm channel height.

At the inlet, a fully developed laminar velocity profile was applied, and the average inlet velocity was set according to the corresponding experimental flow rate for each case. Zero static pressure was set at the outlet, and a pressure point constraint was applied at one node on the outlet cross-section to eliminate the singularity of the pressure field and guarantee a unique solution. No-slip boundary conditions were imposed on all channel walls.

Mesh independence test: The computational domain was discretized using three built-in mesh densities: normal, fine, and finer. The peak vorticity in the contraction region was monitored as the sensitive variable. The results showed that the relative deviation between the fine and finer meshes was below 1%, indicating grid convergence. Therefore, the fine mesh was adopted for all subsequent simulations to balance accuracy and computational efficiency. It should be noted that these simulations are fluid-only and explicitly adopt the same volumetric flow rates as the corresponding experiments (e.g., 200, 300, and 500 μL/min in Section 4.2, Section 4.3 and Section 4.4) to ensure a consistent comparison basis. They are designed to isolate and compare the intrinsic flow-field characteristics induced by the microstructures.

## 4. Results and Discussion

### 4.1. Inertial Migration Characteristics of Particles in Straight Channels

As a control, a straight channel with the same cross-sectional dimensions as the main channel (width *W* = 120 μm and height = 60 μm) and a total length of 24.5 mm was first employed to examine the purely inertial migration behavior of particles with diameters of 5 μm, 7 μm, 10 μm, and 15 μm at a constant flow rate of 200 μL/min. All images were captured at the channel exit.

As shown in Figure 3, the inertial focusing behavior differed markedly among particles of different diameters. The 5 μm and 7 μm particles exhibited a dispersed distribution; the 10 μm particles formed a continuous focusing band in the central region of the channel, although a small number of particles remained near the edges; and the 15 μm particles displayed a significantly narrowed focusing band with a more compact distribution, indicating substantially improved focusing performance.

These observations can be attributed to the fact that only the inertial lift force acts on particles in a straight channel. According to the theoretical scaling relationship $F_{L}\propto U_{m}^{2}a_{p}^{4}$, the inertial lift force is proportional to the square of the flow velocity and the fourth power of the particle diameter. Larger particles experience a stronger inertial lift force and thus focus more rapidly at the channel centerline.

Previous studies have demonstrated that embedding microstructures in a straight channel induces transverse secondary flows [29]. Consequently, the equilibrium positions of particles are governed by the competition between the inertial lift force and secondary flow drag. To elucidate the mechanisms by which microstructures influence particle inertial migration, the following sections systematically analyze the effects of the geometric dimensions of rectangular and hook-shaped microstructures on particle migration behavior.

### 4.2. Comparative Study of Inertial Migration Characteristics in Rectangular and Hook-Shaped Microstructures Under Identical Geometric Parameters

Firstly, numerical simulations were conducted to analyze the flow fields in microchannels embedded with rectangular and hook-shaped microstructures under identical geometric parameters (W=120 μm, H=40 μm, L=500 μm) at an average velocity of 0.46 m/s (corresponding to 200 μL/min), as shown in Figure 4. The presence of the microstructures induces a severe contraction of the fluid. Notably, the sharp right-angle corners of the rectangular microstructures cause a stronger flow separation and steeper local velocity gradients, thereby inducing stronger vortices compared to the streamlined hook-shaped microstructures.

As shown in Figure 5, under identical geometric parameters (*W* = 120 μm, *H* = 40 μm, *L* = 500 μm), the simulation results indicate that the peak vorticity induced by the rectangular microstructures (9.84 × 10^5^ s^−1^) is significantly higher than that induced by the hook-shaped microstructures (4.62 × 10^5^ s^−1^). The vortex area induced by the rectangular microstructures (6.08 × 10^3^ μm^2^) is also larger than that induced by the hook-shaped microstructures (4.89 × 10^3^ μm^2^). A vorticity threshold of 40,000 s^−1^ was applied to identify the vortex regions. This threshold was chosen to exclude low-vorticity background and weak secondary flow, so that only the dominant vortices relevant to particle manipulation are retained; it corresponds to a small proportion of the maximum vorticity (approximately 4–9% of the peak values in the present cases) and serves as the consistent criterion for vortex-region selection in all subsequent simulations. This indicates that, under identical geometric parameters, the rectangular microstructures not only dominate in local pushing strength but also cover a wider effective range within the expansion cavity.

As shown in Figure 6, under the experimental condition of Re = 37 (corresponding to 200 μL/min), 5 μm particles exhibited distinctly different migration behaviors in the two microchannel configurations: particles were stably focused at the channel centerline in the microchannel with hook-shaped microstructures, whereas they clearly migrated toward the sidewalls in the microchannel with rectangular microstructures.

This experimental phenomenon is highly consistent with the simulation results. The simulation analysis (Figure 5) reveals that under identical geometric parameters (*W* = 120 μm, *H* = 40 μm, *L* = 500 μm), the secondary flow intensity (peak vorticity 9.84 × 10^5^ s^−1^) induced by the rectangular microstructures is significantly higher than that of the hook-shaped microstructures (4.62 × 10^5^ s^−1^). Furthermore, the secondary flow area induced by the rectangular microstructures (6.08 × 10^3^ ${\mu m}^{2}$) is also larger than that of the hook-shaped microstructures (4.89 × 10^3^ ${\mu m}^{2}$). Since the magnitude of the secondary flow drag on particles is governed primarily by the local secondary flow intensity, and the length of the acting path is determined by the secondary flow area, the stronger secondary flow intensity and larger secondary flow area in the rectangular channel jointly exert a stronger lateral drag on the particles, forcing the 5 μm particles to leave the centerline and migrate toward the sidewalls. Conversely, the weaker secondary flow intensity and smaller secondary flow area in the hook-shaped channel result in weaker lateral perturbation on the particles, allowing the inertial lift force to dominate and stably focus the particles at the centerline.

The combination of the above experimental and simulation results reveals the significant influence of secondary flow intensity and effective range on particle migration behavior under identical geometric parameters. On this basis, to further refine the governing mechanisms of inertial particle migration in CEA channels, the present study systematically investigates the particle migration behavior in microchannels embedded with rectangular and hook-shaped microstructures under varying embedding depths (*H*) and expansion segment lengths (*L*).

### 4.3. Inertial Migration Characteristics of Particles in Contraction-Expansion Channels Embedded with Rectangular Microstructures

Under simulation conditions with a main channel width W=120 μm and a height of 60 μm, we systematically investigated the effects of embedding depth (H) and expansion segment length (L) on the secondary flow characteristics in rectangular microchannels. Specifically, the simulations for the embedding depth (H) were performed at an average velocity of approximately 0.7 m/s (corresponding to 300 μL/min), whereas the simulations for the expansion segment length (L) were conducted at an average velocity of approximately 1.16 m/s (corresponding to 500 μL/min).

To quantitatively analyze the regulatory effects of geometric parameters on secondary flow characteristics, numerical simulations were first performed to obtain the flow field distributions of rectangular microstructures under varying embedding depths (*H*) and expansion segment lengths (*L*) (Figure 7). The corresponding peak vortex intensity and vortex area were subsequently extracted (Figure 8 and Figure 9).

The flow field simulation results (Figure 7a) intuitively demonstrate that the increase in embedding depth significantly alters the flow structure. The vorticity threshold was set to 70,000 s^−1^. The quantitative data (Figure 8) further confirm that as *H* increases from 30 μm to 40 μm, the secondary flow intensity (peak vorticity) significantly increases from 1.02 × 10^6^ s^−1^ to 2.14 × 10^6^ s^−1^, while the vortex area expands from 2.33 × 10^3^ μm^2^ to 5.13 × 10^3^ μm^2^, both increasing by more than 100%. This indicates that increasing the embedding depth can significantly enhance both the local intensity and effective range of the secondary flow.

In contrast, as shown in the quantitative results of Figure 9, the expansion segment length *L* exhibits an extremely limited effect on the secondary flow characteristics, with a uniform vorticity threshold of 120,000 s^−1^ applied for the vortex area extraction, as *L* increases from 100 μm to 400 μm, the secondary flow intensity only fluctuates slightly within a range from 2.18 × 10^6^ s^−1^ to 1.93 × 10^6^ s^−1^, and the vortex area remains essentially stable within the narrow range of 2.23 × 10^3^ μm^2^ to 2.13 × 10^3^ μm^2^, neither showing significant variations. This indicates that the secondary flow intensity and effective range are insensitive to variations in the expansion segment length, remaining relatively stable within a certain range.

Based on the numerical simulation results (Figure 7, Figure 8 and Figure 9), for rectangular microstructures, the embedding depth *H* is significantly positively correlated with the secondary flow intensity and effective range. As *H* increases from 30 μm to 40 μm, the peak vorticity increases from 1.02 × 10^6^ s^−1^ to 2.14 × 10^6^ s^−1^, and the vortex area expands from 2.33 × 10^3^ μm^2^ to 5.13 × 10^3^ μm^2^. In contrast, the expansion segment length *L* exhibits an extremely limited influence on the secondary flow characteristics (Figure 9), indicating that the secondary flow intensity and effective range are insensitive to variations in *L* and remain relatively stable. Therefore, the embedding depth serves as the key parameter regulating the secondary flow effect.

Figure 10 illustrates the influence of the embedding depth H on particle inertial migration behavior, with the expansion segment length fixed at L=400 μm. According to the simulation results (Figure 7 and Figure 8), increasing H from 30 μm to 40 μm significantly enhances both the secondary flow intensity (peak vorticity increases from $1.02\times 10^{6}\;s^{-1}$ to $2.14\times 10^{6}\;s^{-1}$) and its effective range (vortex area expands from $2.33\times 10^{3}\;\mu m^{2}$ to $5.13\times 10^{3}\;\mu m^{2}$). At a flow rate of 300 μL/min (Re = 55.6) (Figure 10a), as H increases from 30 μm to 40 μm, the intensified secondary flow drag drives the 7 μm particles toward the channel sidewalls, whereas the 15 μm particles remain focused at the channel centerline due to the stronger inertial lift force.

At a flow rate of 800 μL/min (Re = 148.2) (Figure 10b), the 15 μm particles focus at the channel centerline when H=30 μm. This behavior is similar to that observed at H=40 μm and 300 μL/min, with both cases dominated by the inertial lift force. Notably, the lower flow rate required to achieve centerline focusing at a larger embedding depth indicates that increasing H can reduce the flow rate needed for stable centerline focusing of 15 μm particles, thereby enhancing focusing efficiency under inertial lift dominance. However, the combination of a larger embedding depth and high flow rate amplifies the secondary flow effect, causing some particles to be temporarily entrained or retained within the vortex regions. This behavior is observed only as a projected lateral distribution; no residence-time or three-dimensional trapping analysis was performed.

These results demonstrate that the embedding depth H of rectangular microstructures is positively correlated with secondary flow intensity and effective range. Increasing H drives the 7 μm particles toward the sidewalls under enhanced secondary flow drag while simultaneously improving the centerline focusing efficiency of the 15 μm particles. The coupling of a high flow rate and a large embedding depth can lead to temporary particle entrainment within the vortex regions.

Figure 11 illustrates the influence of the expansion segment length L on particle inertial migration behavior. Throughout these experiments, the embedding depth was fixed at H=30 μm. Consistent with the simulation results, the secondary flow intensity and effective range are insensitive to variations in L. At a flow rate of 100 μL/min (Re = 18.5) (Figure 11a), as L increases from 100 μm to 400 μm, the particle migration behavior is primarily governed by the subtle adjustment of the competition between the inertial lift force and the secondary flow drag. Since the secondary flow range remains relatively stable, 7 μm particles can still maintain lateral migration in shorter expansion segments, but their equilibrium state is slightly disturbed as L increases due to the relatively weakened secondary flow perturbation. Meanwhile, the 15 μm particles exhibit a hybrid migration behavior combining centerline focusing and lateral dispersion across all three expansion segment lengths.

When the flow rate was increased to 500 μL/min (Re = 92.6) (Figure 11b), the migration pattern of the 7 μm particles remained essentially unchanged. In contrast, the 15 μm particles displayed a notable transition: as the expansion segment length increased, the secondary flow perturbation remained relatively stable, allowing the inertial lift force to gradually dominate and drive the 15 μm particles to migrate toward the channel centerline and achieve stable inertial focusing.

These results demonstrate that, for rectangular microstructures, increasing the embedding depth H simultaneously enhances both the intensity and effective range of the secondary flow, thereby promoting particle migration toward the sidewalls under the effect of the secondary flow. When H is fixed, once the microstructure-induced vortices have fully developed within the expansion cavity, further increasing L weakens the effect of the secondary flow, thereby allowing the inertial lift force to become dominant, especially for larger particles such as 15 μm.

### 4.4. Inertial Migration Characteristics of Particles in Contraction-Expansion Channels Embedded with Hook-Shaped Microstructures

Under simulation conditions with a main channel width of 180 μm and a height of 60 μm, we systematically investigated the effects of embedding depth (H) and expansion segment length (L) on the secondary flow characteristics in hook-shaped microstructures. For the investigation of the embedding depth H, the expansion segment length was fixed at L=400 μm, and the average velocity was 0.46 m/s (corresponding to 300 μL/min). For the investigation of the expansion segment length L, the embedding depth was fixed at H=60 μm, and the average velocity was 0.77 m/s (corresponding to 500 μL/min). Figure 12 intuitively illustrates the flow field distributions of hook-shaped microstructures under different geometric parameters. As shown in Figure 12a, when the expansion segment length is fixed at L=400 μm, increasing the embedding depth H from 50 μm to 70 μm significantly intensifies the flow perturbation, making the vortex structures more pronounced.

The quantitative results in Figure 13 further confirm this trend: with a uniform vorticity threshold of 30,000 s^−1^ applied for the vortex area extraction, the peak vortex intensity increases from $2.59\times 10^{5}\;s^{-1}$ to $6.68\times 10^{5}\;s^{-1}$, and the vortex area expands from $4.94\times 10^{3}\;\mu m^{2}$ to $11.80\times 10^{3}\;\mu m^{2}$, both exceeding a 100% increase. This indicates that increasing the embedding depth can significantly enhance both the local intensity and effective range of the secondary flow.

In contrast, the influence of the expansion segment length L is different. As shown in Figure 12b, when the embedding depth is fixed at H=60 μm, as L increases from 100 μm to 400 μm, the flow field structure does not change dramatically, but the spatial extension of the vortices is noticeably enlarged. The quantitative results in Figure 14 clearly reveal this trend: with a uniform vorticity threshold of 40,000 s^−1^ applied for the vortex area extraction, the peak vortex intensity only fluctuates slightly from $7.55\times 10^{5}\;s^{-1}$ to $7.43\times 10^{5}\;s^{-1}$, remaining essentially stable; whereas the vortex area continuously increases from $7.23\times 10^{3}\;\mu m^{2}$ to $13.65\times 10^{3}\;\mu m^{2}$, nearly doubling. This demonstrates that in hook-shaped microstructures, increasing the expansion segment length cannot significantly enhance the secondary flow intensity, but it effectively expands its effective range.

The simulation results (Figure 13) reveal that, for the hook-shaped microstructures, increasing the embedding depth H from 50 μm to 70 μm simultaneously enhances the secondary flow intensity (peak vorticity increasing from $2.59\times 10^{5}\;s^{-1}$ to $6.68\times 10^{5}\;s^{-1}$) and its effective range (vortex area expanding from $4.94\times 10^{3}\;\mu m^{2}$ to $11.80\times 10^{3}\;\mu m^{2}$). These simulation findings are well corroborated by the experimental observations in Figure 15. At a flow rate of 300 μL/min and with the expansion segment length fixed at L=400 μm, the motion of the 7 μm particles is consistently dominated by the secondary flow drag. As H increases, the enhanced secondary flow drives these particles more stably toward equilibrium positions near the channel sidewalls, resulting in a markedly narrowed focusing band and significantly improved focusing performance.

For the 15 μm particles, the competition between the inertial lift force and the secondary flow drag leads to two distinct migration modes as H increases. At H=50 μm, the particles form a relatively broad focused stream along the channel centerline. When H is increased to 60 μm, this stream narrows, indicating a strengthened dominance of the inertial lift force. Upon further increasing H to 70 μm, the secondary flow drag becomes dominant, causing the focused band to split toward the sidewalls and ultimately form two separate streams. These two migration behaviors originate from the competition between the inertial lift force and the secondary flow drag: when the inertial lift force dominates, particles focus toward the centerline; when the secondary flow drag prevails, particles are pushed toward the sidewalls. In contrast to the 15 μm particles, the 7 μm particles, owing to their smaller diameter, exhibit sidewall-directed migration governed by the secondary flow at all embedding depths.

The simulation results (Figure 14) reveal that, for the hook-shaped microstructures with the embedding depth fixed at H=60 μm, increasing the expansion segment length L from 100 μm to 400 μm causes only a slight fluctuation in the secondary flow intensity (peak vorticity from $7.55\times 10^{5}\;s^{-1}$ to $7.43\times 10^{5}\;s^{-1}$), which remains essentially stable; whereas the vortex area significantly increases from $7.23\times 10^{3}\;\mu m^{2}$ to $13.65\times 10^{3}\;\mu m^{2}$, nearly doubling. This indicates that in hook-shaped microstructures, increasing L has only an extremely limited effect on the secondary flow intensity but effectively expands its effective range.

This simulation trend is highly consistent with the experimental observations in Figure 15. At a flow rate of 500 μL/min (Re = 69.4) (Figure 16a), as the expansion segment length increases, the effective range of the secondary flow continuously expands, progressively driving the 7 μm particles away from the channel centerline toward equilibrium positions near the sidewalls. Meanwhile, the 15 μm particles gradually focus toward the channel centerline; at L=400 μm, they are completely focused at the centerline. However, when L is further increased to 500 μm, the equilibrium state of the 15 μm particles is disrupted under the influence of the secondary flow, causing the focused band to disperse toward the sidewalls.

When the flow rate is elevated to 800 μL/min (Re = 111.1) (Figure 16b), the combination of high flow velocity and large expansion segment length further enhances both the intensity and effective range of the secondary flow. Under these conditions, both the 7 μm and 15 μm particles exhibit pronounced lateral dispersion and are temporarily entrained within the vortex regions in the projected images. No stable three-dimensional trapping is claimed, as the vertical coordinate was not resolved in the present study.

These results indicate that, for the hook-shaped microstructures, increasing the embedding depth H enhances both the intensity and effective range of the secondary flow, which is similar to the effect observed when H is increased in the rectangular microstructures. However, when H is fixed, the secondary flow vortices induced by the hook-shaped microstructures become fully developed as the expansion segment length L increases, and it can be anticipated that the effective range of the secondary flow acting on particles will be further enlarged.

Based on the experimental results, a hook-shaped contraction–expansion array (CEA) channel with a width w=180 μm, an expansion segment length L=400 μm, and a channel height of 60 μm was selected for the particle separation experiments. A trifurcated outlet with three branches of equal width (ratio 1:1:1) was positioned at the channel terminus. The structural parameters of the branched outlet are shown in Figure 2. A 1 mL mixed suspension was prepared, containing 7 μm particles at a concentration of $1.5\times 10^{5}$ particles/mL and 15 μm particles at $5\times 10^{4}$ particles/mL, and was thoroughly vortexed for 3 min prior to experiments. The mixed suspension was infused into the channel inlet at a flow rate of 500 μL/min. The particle trajectory distribution at the outlets is shown in Figure 17a: the 7 µm particles exited through the side outlets, whereas the 15 μm particles, dominated by the inertial lift force, exited through the center outlet. The definitions of purity and recovery rate are provided in Section 3.2.3.

As shown in Figure 17b, the grayscale value distribution across the channel width further quantifies the particle positions at the outlets. Two distinct grayscale peaks corresponding to the 7 μm particles appear near the side outlets, while one grayscale peak corresponding to the 15 μm particles appears near the center outlet. This distribution is consistent with the trajectory results in Figure 17a, confirming that the 7 μm and 15 μm particles are clearly separated at the outlets.

As shown in Figure 17c, based on the particle counts in the solutions collected from each outlet, the purity and recovery rate of the 7 μm particles at the side outlets were 98.5±0.25% and 99.1±0.26%, respectively, whereas those of the 15 μm particles at the center outlet were 98.7±0.17% and 99.3±0.10%. For the side outlets, the effluents from the two side branches were pooled and counted together for the 7 μm particles. Data are presented as mean ± standard deviation (SD) from three independent experiments (n=3), and the error bars in Figure 17c represent the SD. These results demonstrate that the hook-shaped microchannel enables highly efficient size-based inertial separation.

## 5. Conclusions

This study combines numerical simulations and experiments to systematically compare the modulation of particle inertial migration by geometric parameters in contraction–expansion array (CEA) channels embedded with rectangular and hook-shaped microstructures, and to elucidate the configuration-dependent competition among inertial lift, secondary flow drag, and the effective range of the secondary flow. For both channel types, a larger embedding depth H simultaneously enhances the peak secondary flow intensity and enlarges its effective range, thereby promoting particle migration toward the channel sidewalls. In contrast, the secondary flow intensity is only weakly sensitive to the expansion segment length L. In rectangular microstructures, the secondary flow is already fully developed within short expansion cavities; further increasing L does not enlarge the effective range but instead weakens the modulation effect of the secondary flow on particles, allowing inertial lift to become more prominent. In hook-shaped microstructures, the secondary flow is not fully developed in short cavities, and increasing L promotes its development and markedly enlarges its effective range, thereby strengthening lateral particle regulation. Once the secondary flow is fully developed, its effective range saturates, and further increasing L similarly weakens the modulation effect of the secondary flow, allowing inertial lift to dominate again. Using the experimental structure, efficient label-free inertial separation of 7 μm and 15 μm particles was achieved, with a recovery rate of 99.3% for 15 μm particles and both purity and recovery exceeding 98%. This work elucidates the key role of the effective range of the secondary flow in inertial particle migration and provides theoretical guidance for the rational design of CEA-based microfluidic devices, with potential applications in high-throughput size-based sorting. Finally, it should be noted that the present experiments are based on top-view bright-field imaging, which resolves only the projected lateral particle distributions. The vertical coordinate and three-dimensional equilibrium positions were not resolved. Therefore, the conclusions of this work are limited to the projected lateral migration behavior, and no claims are made about stable three-dimensional trapping. Future work using depth-resolved imaging techniques will be needed to further validate the three-dimensional equilibrium states.

## Figures and Tables

**Figure 1:**
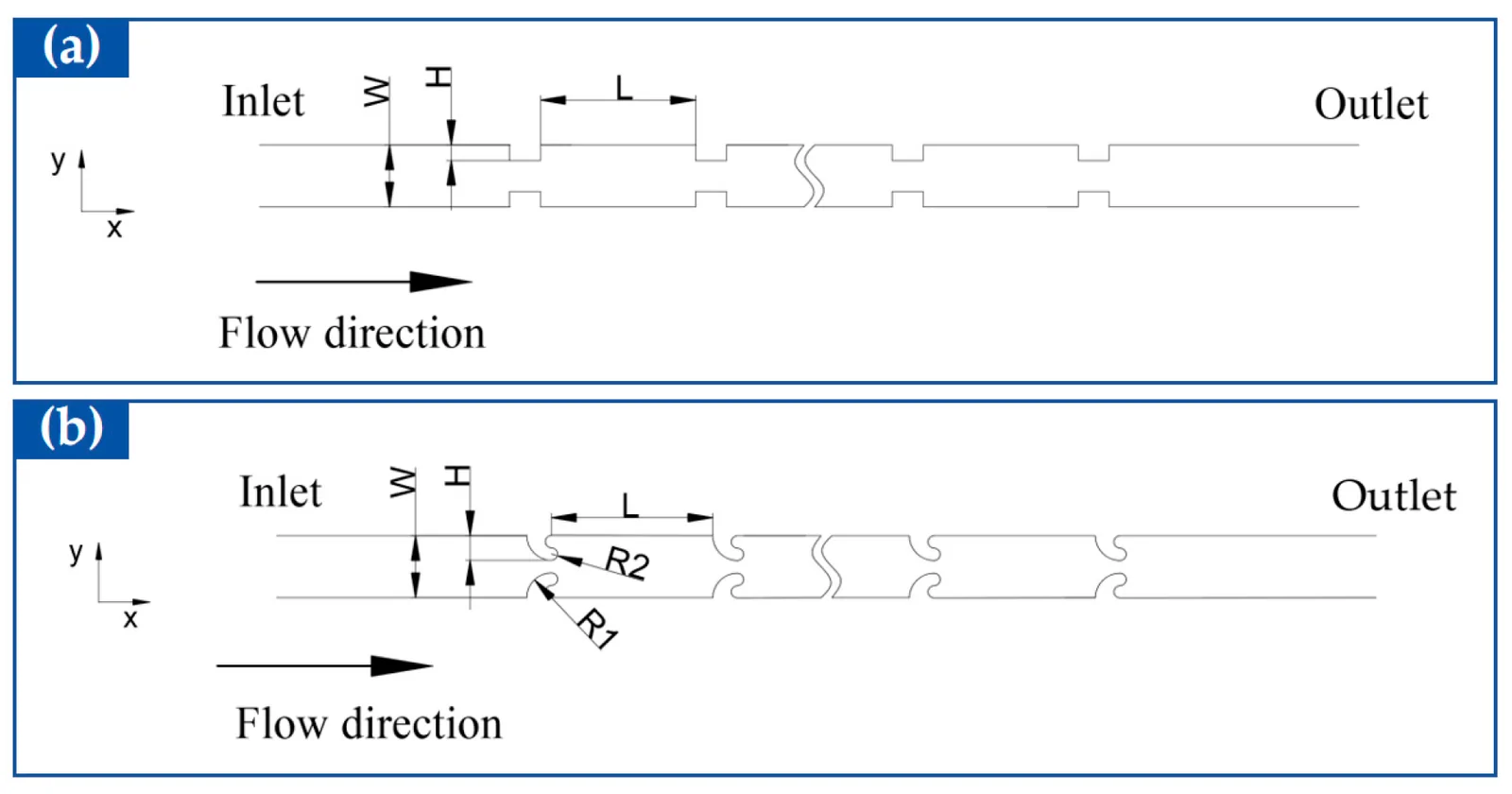
Schematic illustration of the microchannels: (**a**) rectangular microstructures and (**b**) hook-shaped microstructures.

**Figure 2:**
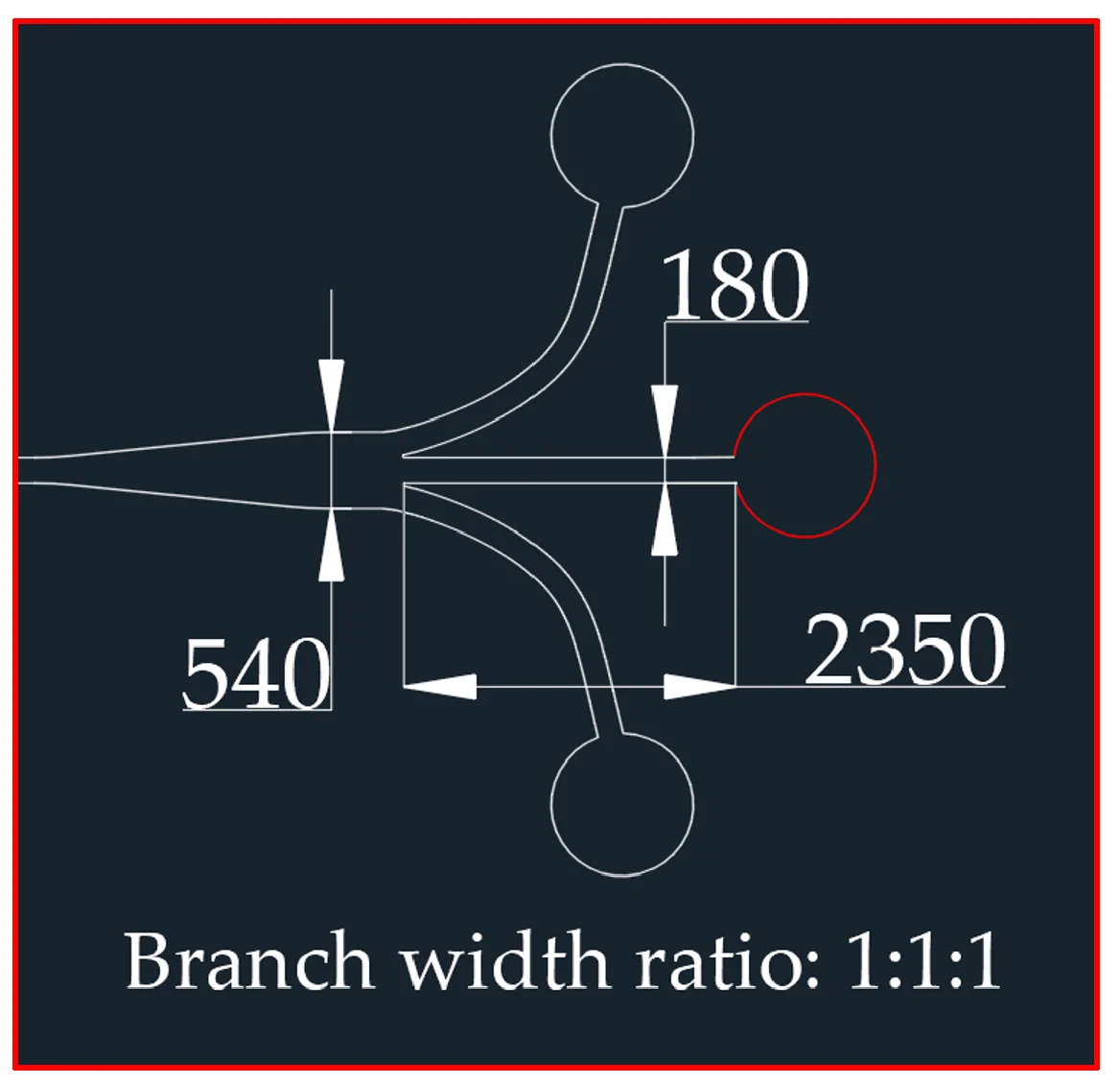
Schematic of the trifurcated outlet structure. The main channel width is 540 μm, and the three outlet branches are equal in width, each being 180 μm (branch width ratio = 1:1:1).

**Figure 3:**
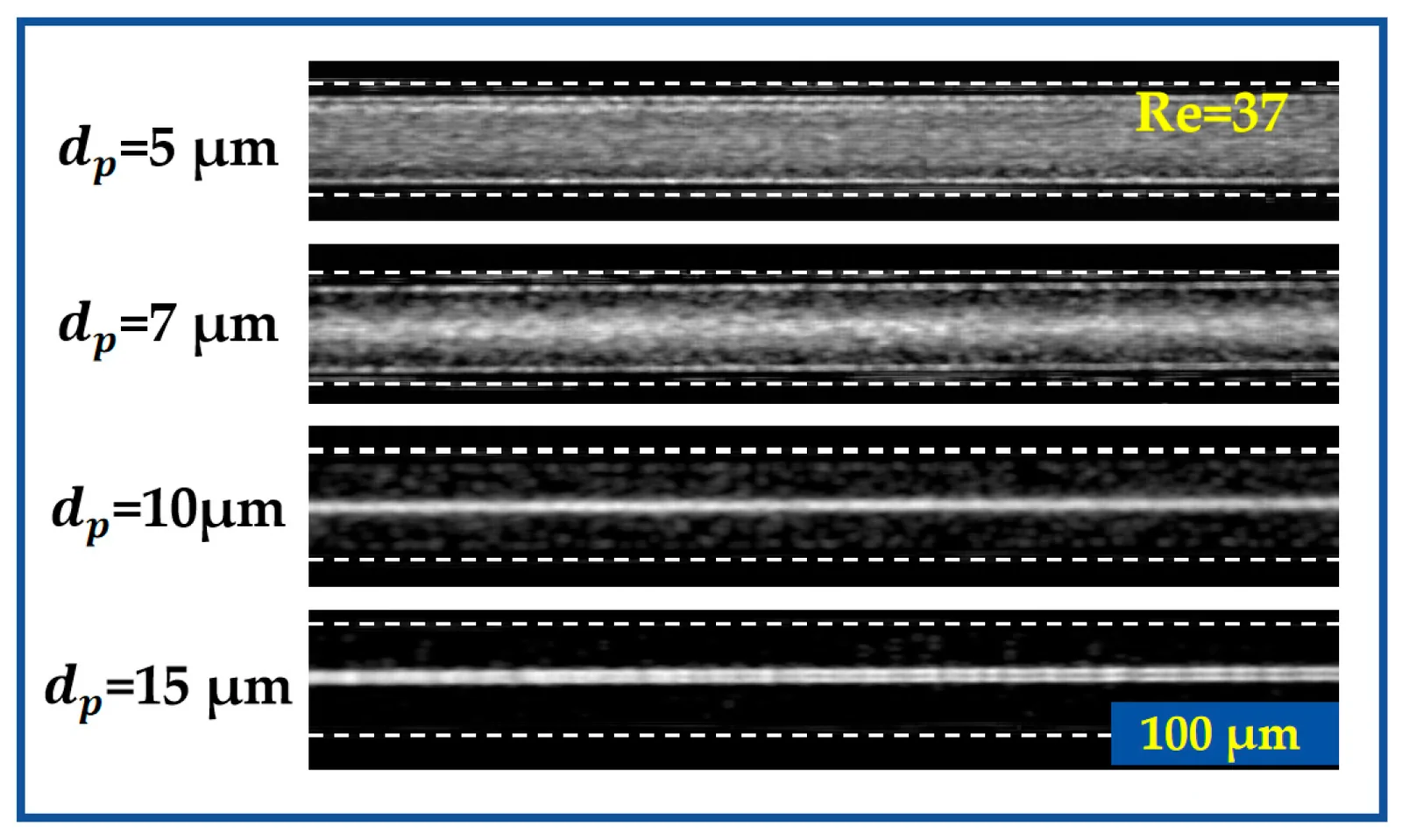
Inertial migration behavior of 5 µm, 7 µm, 10 µm, and 15 µm particles in a straight microchannel.

**Figure 4:**
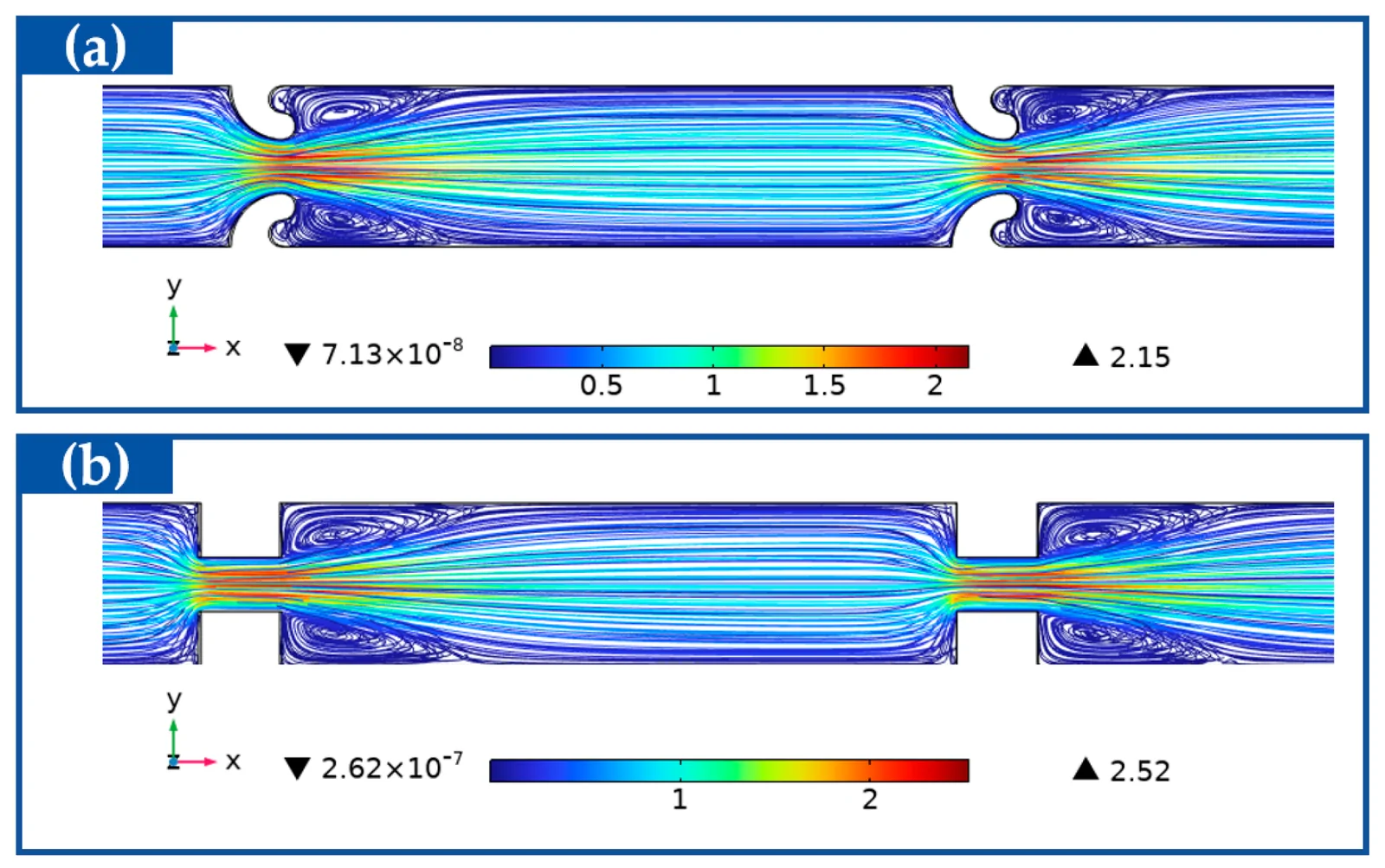
Comparison of velocity fields and streamlines in microchannels embedded with (**a**) rectangular and (**b**) hook-shaped microstructures under identical geometric parameters (*W* = 120 μm, *H* = 40 μm, *L* = 500 μm). The color bar represents the velocity magnitude (m/s).

**Figure 5:**
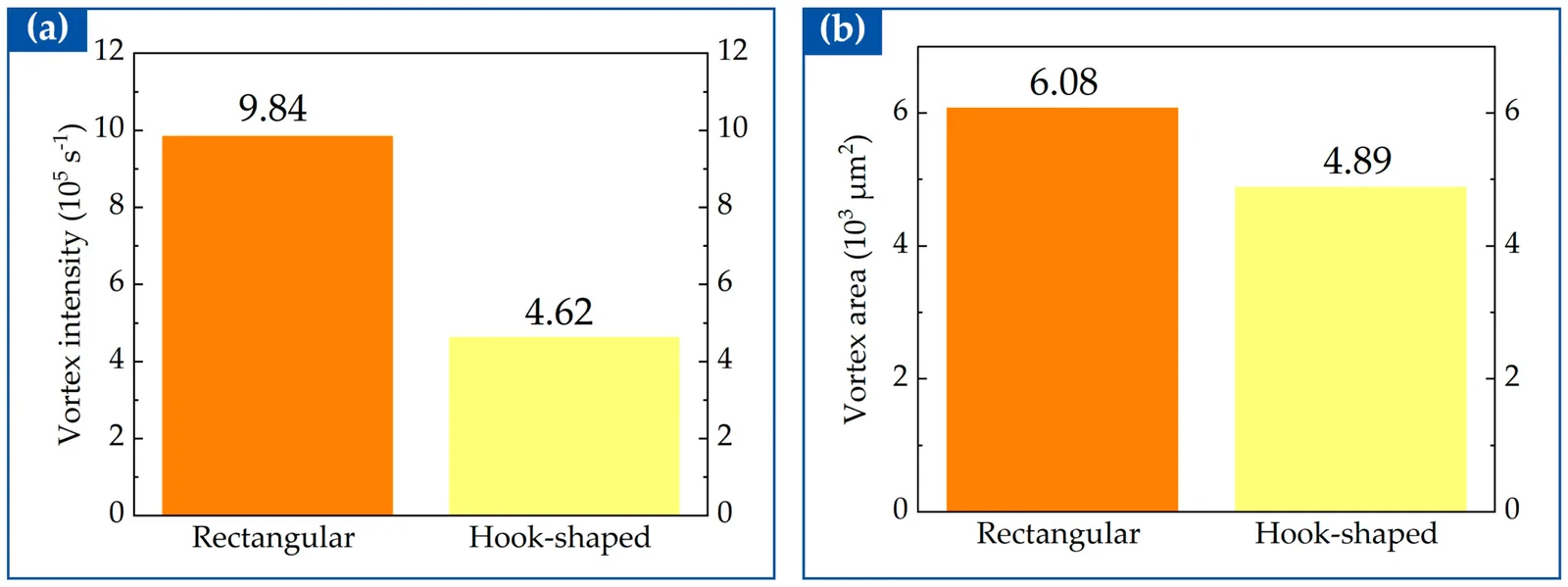
Quantitative comparison of secondary flow vortex characteristics induced by rectangular and hook-shaped microstructures under identical geometric parameters (*W* = 120 μm, *H* = 40 μm, *L* = 500 μm): (**a**) peak vortex intensity; (**b**) vortex area.

**Figure 6:**
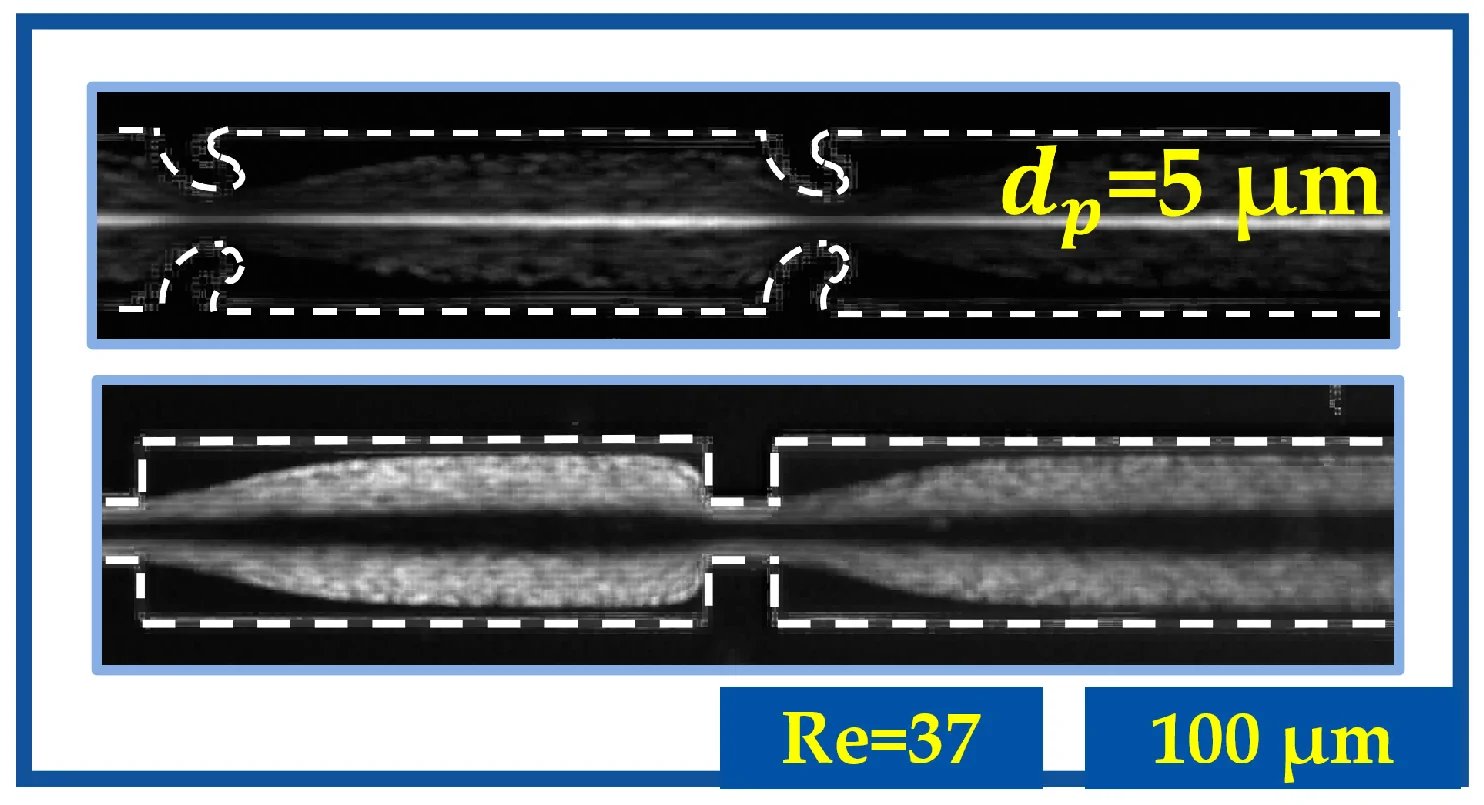
Comparison of the inertial migration behaviors of 5 μm particles in microchannels embedded with hook-shaped and rectangular microstructures at Re = 37 (corresponding to 200 μL/min). Particles are stably focused at the channel centerline in the hook-shaped microchannel, whereas they clearly migrate toward the sidewalls in the rectangular microchannel.

**Figure 7:**
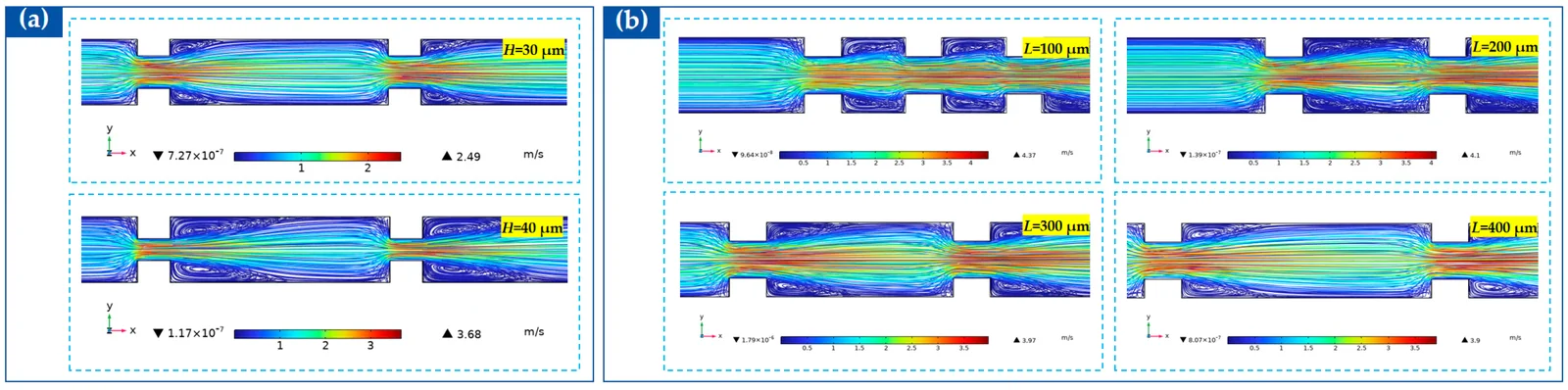
Effects of the embedding depth (H) and expansion segment length (L) on the flow field distributions for rectangular microstructures. (**a**) With the expansion segment length fixed at L=400 μm, comparison of the flow field distributions for H=30 μm and H=40 μm. (**b**) With the embedding depth fixed at H=30 μm, comparison of the flow field distributions for L=100,200,300,and 400 μm. The color bar represents the velocity magnitude (m/s).

**Figure 8:**
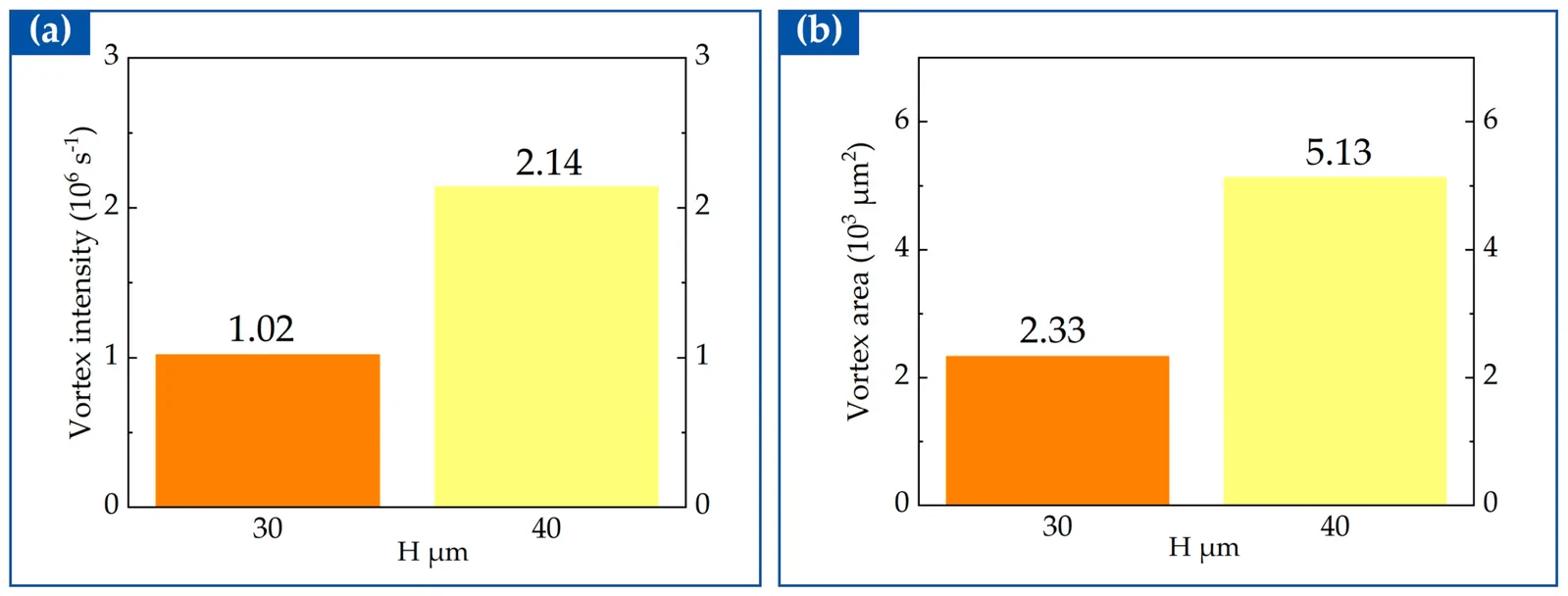
Quantitative characterization of the effects of embedding depth (*H*) on secondary flow characteristics in rectangular microstructures: (**a**) peak vortex intensity; (**b**) vortex area.

**Figure 9:**
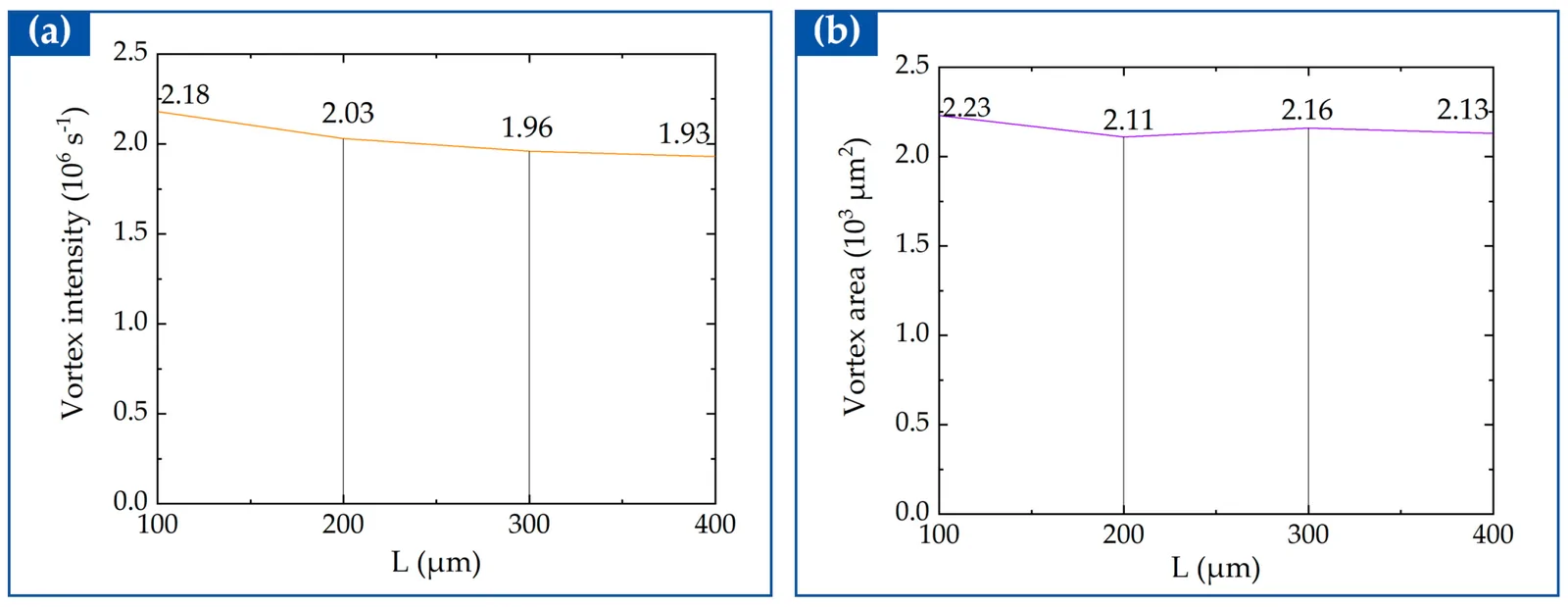
Quantitative characterization of the effects of expansion segment length (*L*) on secondary flow characteristics in rectangular microstructures: (**a**) peak vortex intensity; (**b**) vortex area.

**Figure 10:**
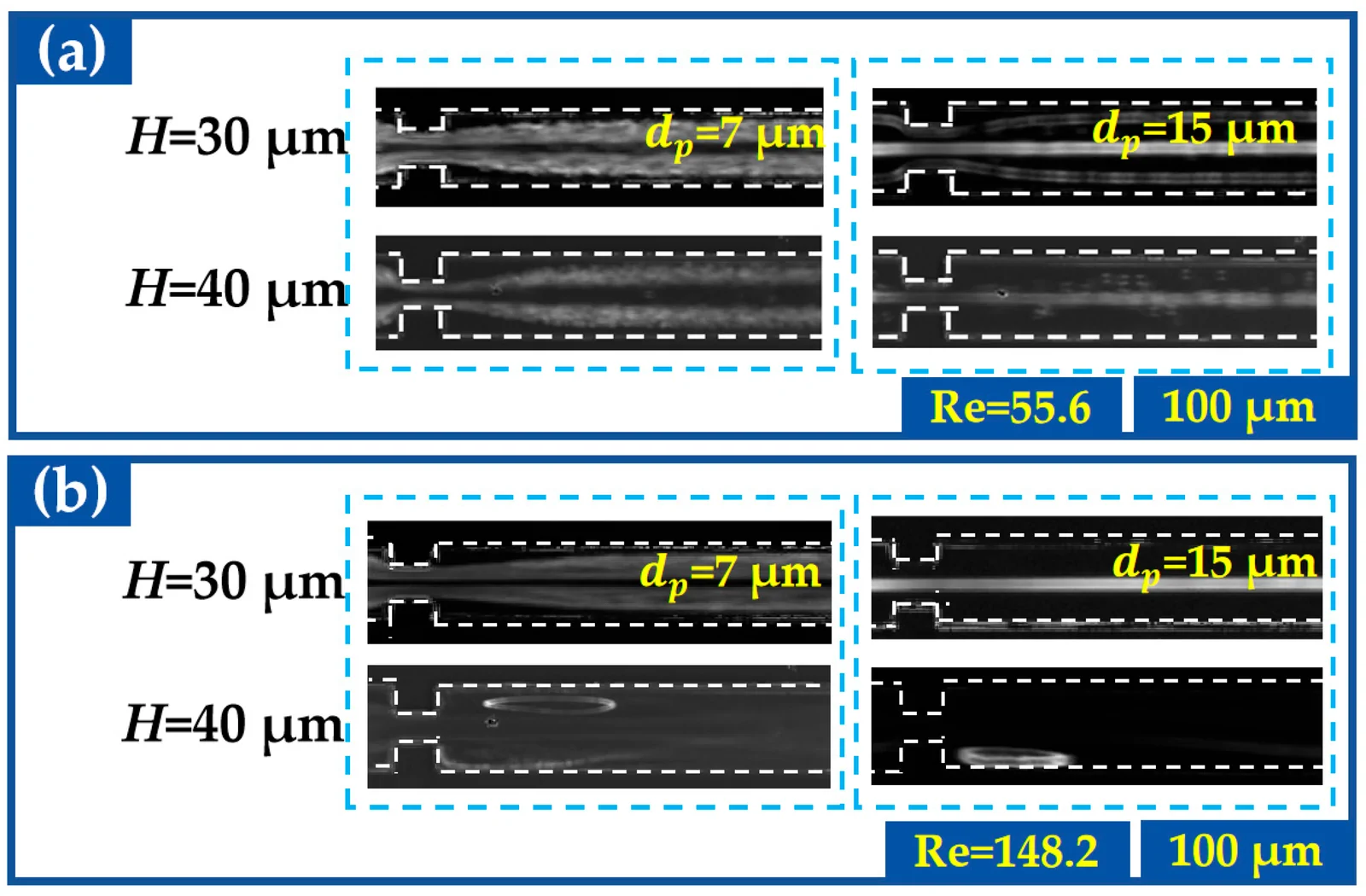
Influence of the embedding depth H of rectangular microstructures on the inertial migration behaviors of 7 µm and 15 µm particles, with the expansion segment length fixed at L=400 μm. (**a**) Particle migration trajectories at a flow rate of 300 µL/min; (**b**) particle migration trajectories at a flow rate of 800 µL/min.

**Figure 11:**
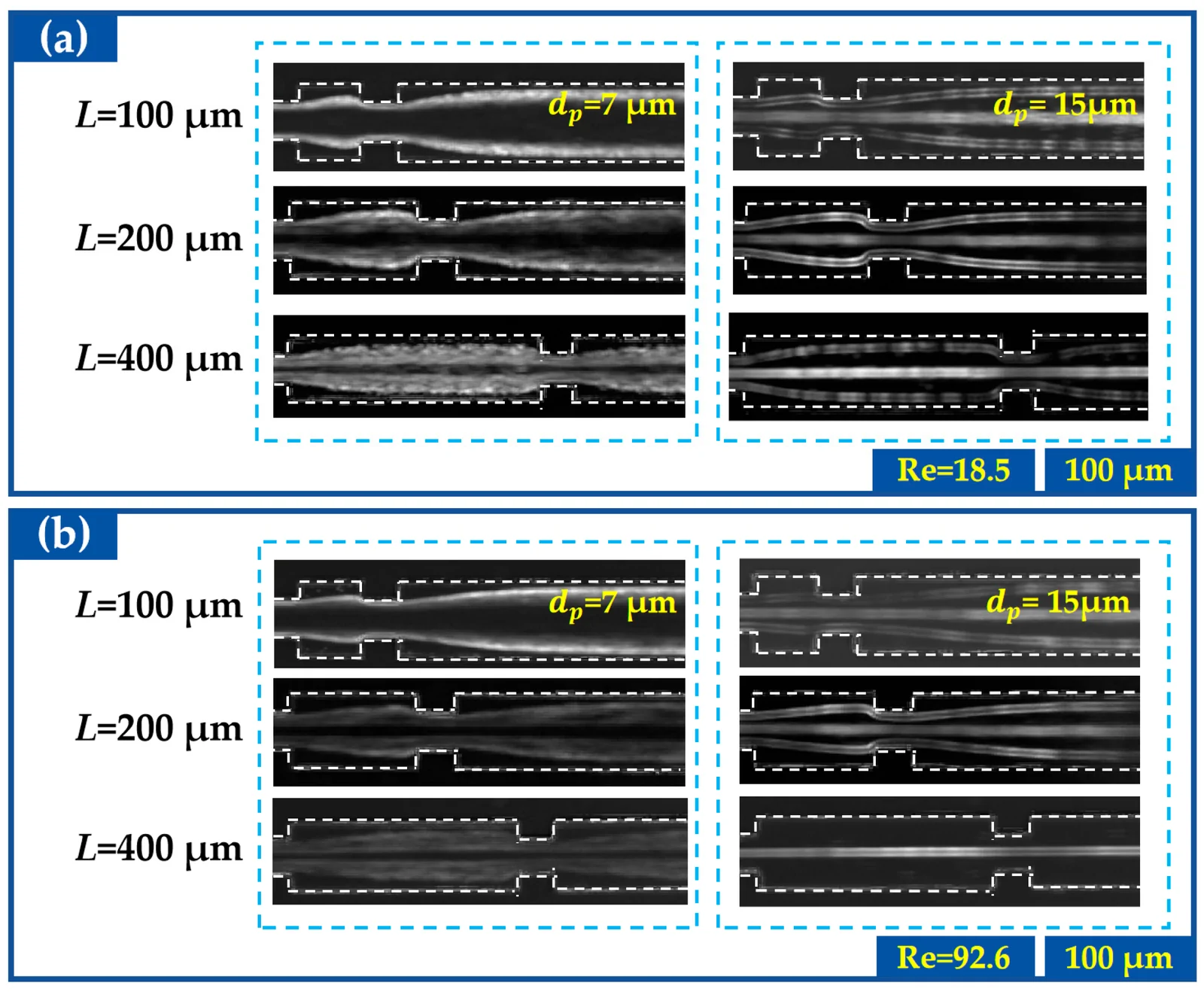
Effect of the expansion segment length *L* on the inertial migration behaviors of 7 µm and 15 µm particles with the embedding depth fixed at H=30 μm. (**a**) Migration trajectories of particles at different *L* with a flow rate of 100 µL/min; (**b**) migration trajectories at a flow rate of 500 µL/min, demonstrating that a larger *L* facilitates stable center focusing of 15 µm particles at high flow rates.

**Figure 12:**
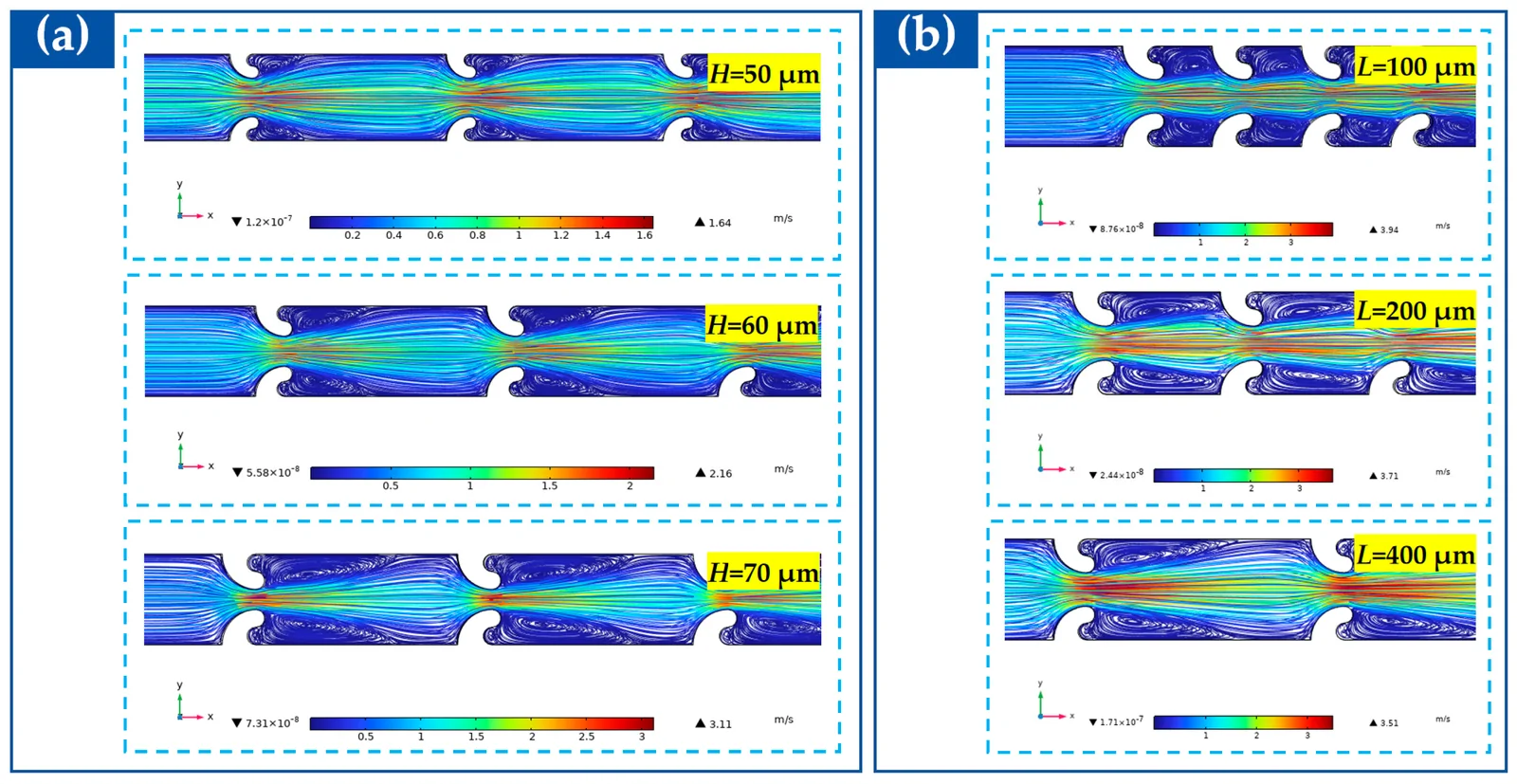
Effects of the embedding depth H and expansion segment length L on the flow field for hook-shaped microstructures. (**a**) With the expansion segment length fixed at L=400 μm, comparison of flow fields for different embedding depths (H=50, 60, and 70 μm). (**b**) With the embedding depth fixed at H=60 μm, comparison of flow fields for different expansion segment lengths (L=100, 200, and 400 μm). The color bar represents the velocity magnitude (m/s).

**Figure 13:**
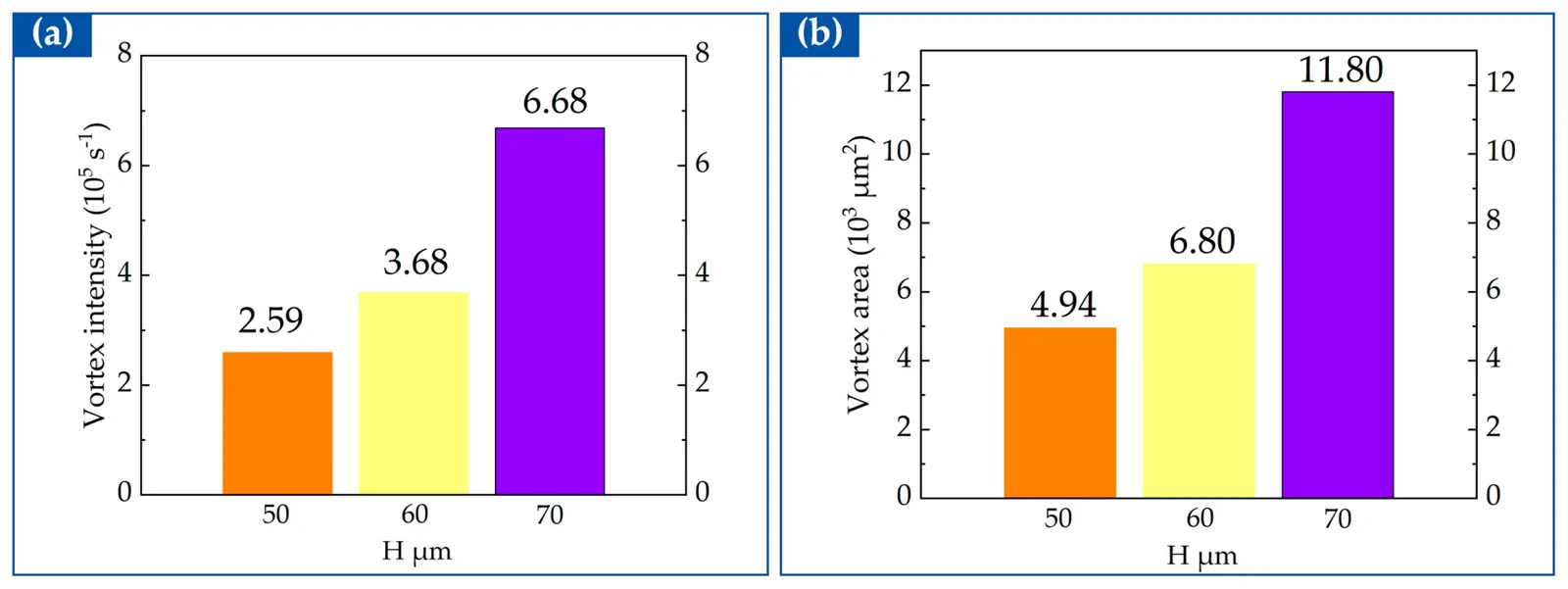
Quantitative characterization of the effects of embedding depth (H) on secondary flow characteristics in hook-shaped microstructures (with the expansion segment length fixed at L=400 μm): (**a**) peak vortex intensity; (**b**) vortex area.

**Figure 14:**
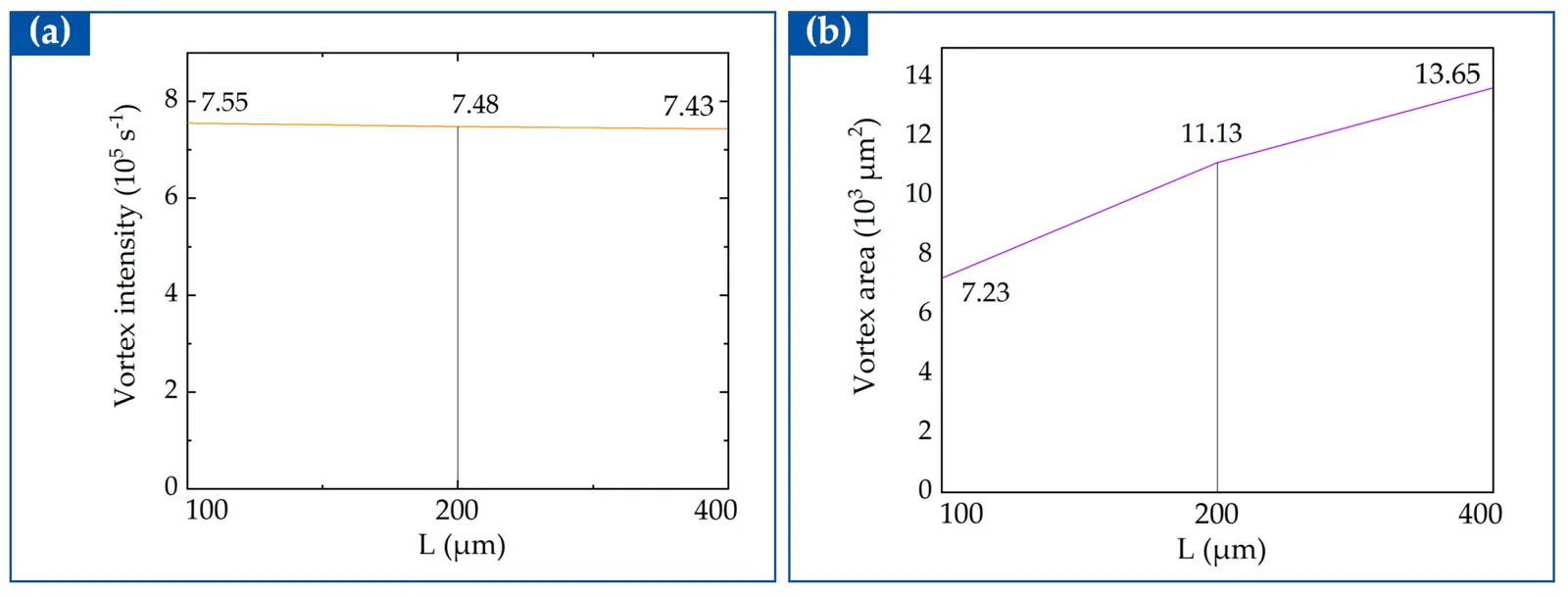
Quantitative characterization of the effects of expansion segment length (L) on secondary flow characteristics in hook-shaped microstructures (with the embedding depth fixed at H=60 μm): (**a**) peak vortex intensity; (**b**) vortex area.

**Figure 15:**
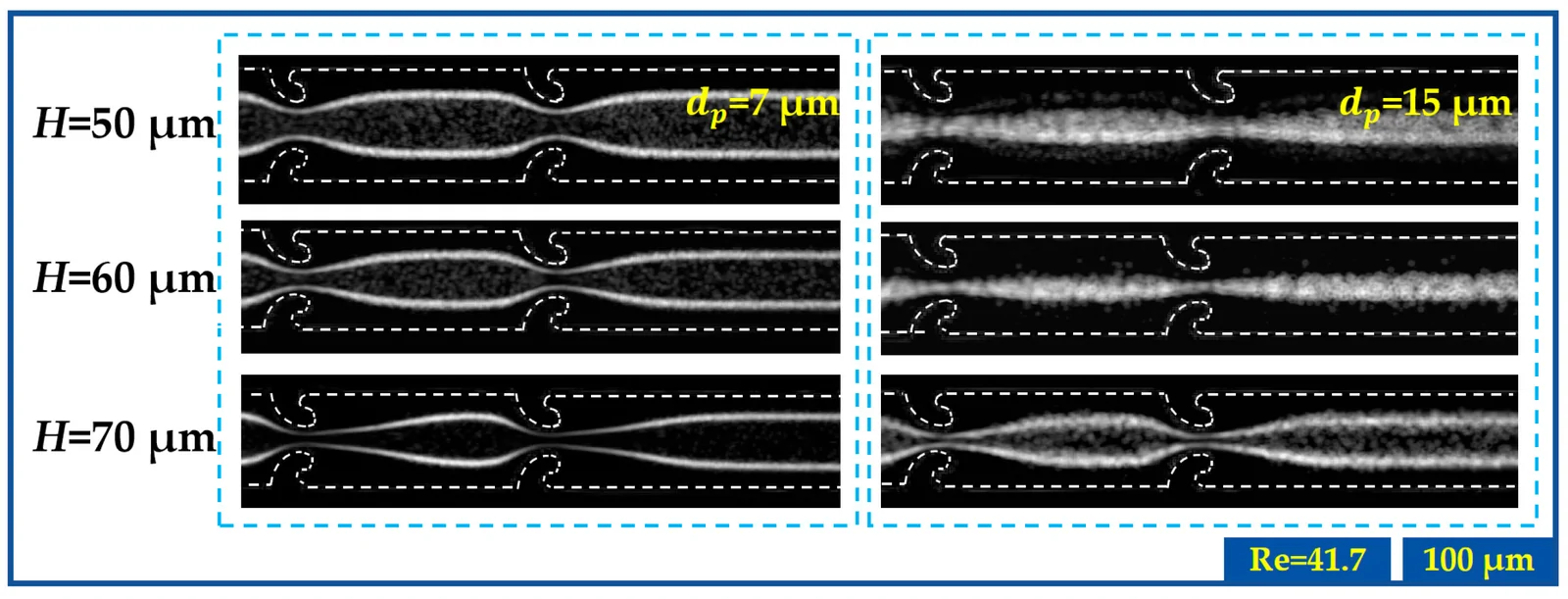
Effect of the embedding depth H on the inertial migration behaviors of 7 μm and 15 μm particles in the hook-shaped microchannel, with the expansion segment length fixed at L=400 μm and a flow rate of 300 μL/min (Re = 41.7).

**Figure 16:**
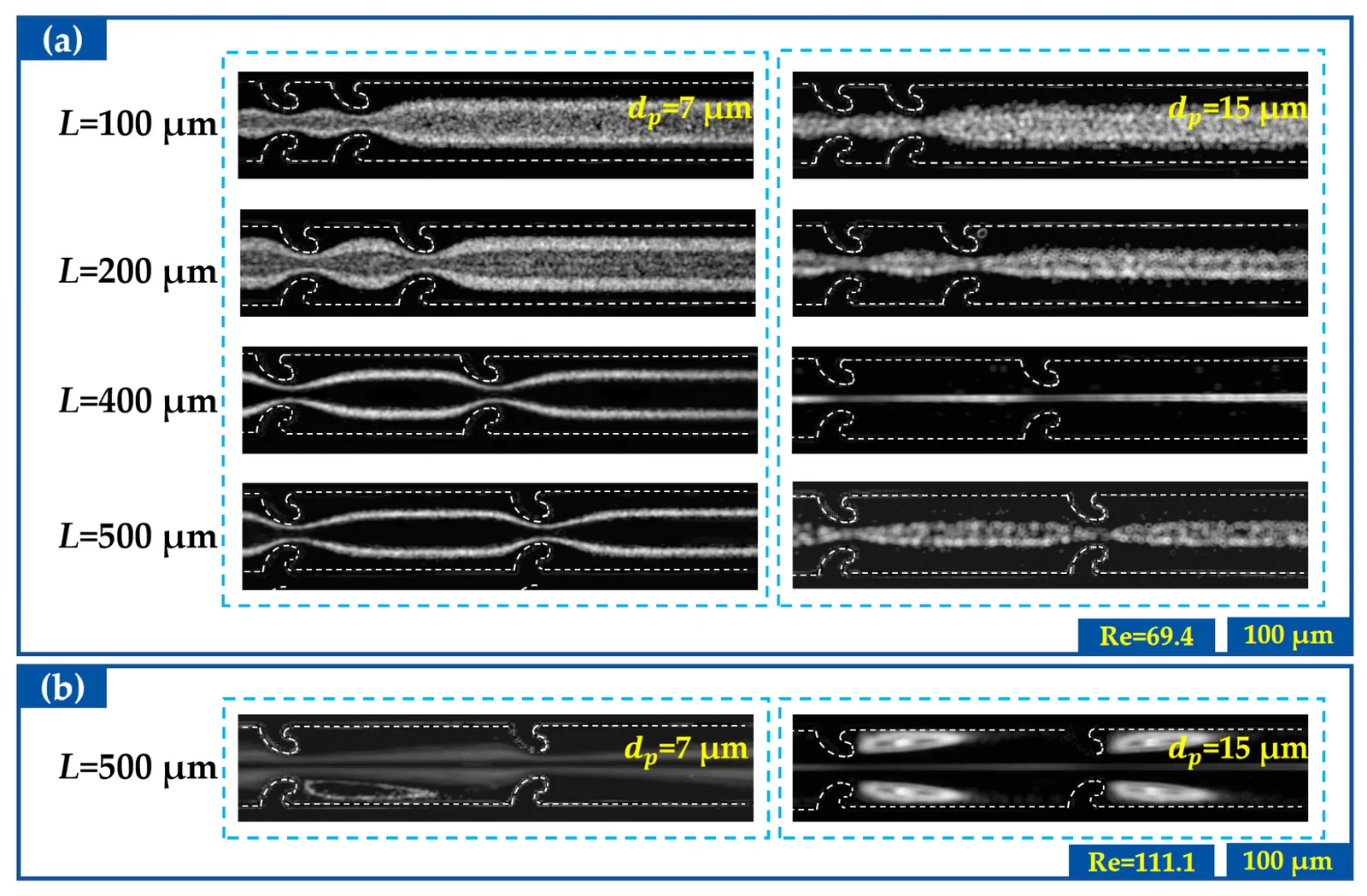
Effect of the expansion segment length L on the migration behaviors of 7 µm and 15 µm particles in the channel with hook-shaped microstructures, with the embedding depth fixed at H=60 μm: (**a**) particle trajectories at different L values with a flow rate of 500 μL/min, showing the transition behavior of 15 µm particles from centerline focusing to lateral dispersion as L increases; (**b**) particle trajectories at a flow rate of 800 μL/min (L=500 μm).

**Figure 17:**
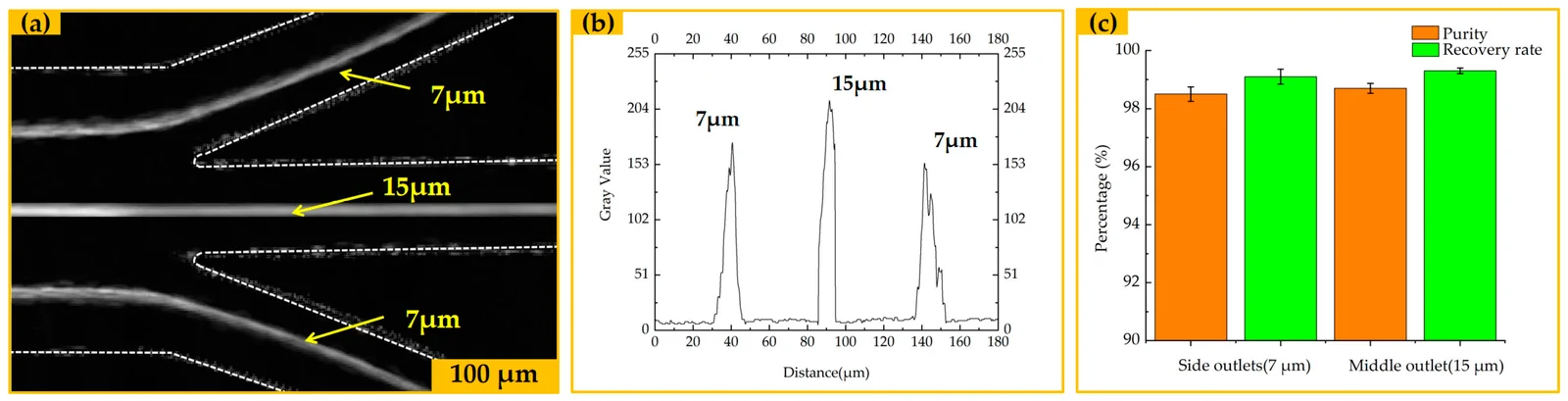
Experimental results of 7 μm and 15 μm particle separation in the hook-shaped CEA channel (W=180 μm,H=60 μm, L=400 μm, flow rate = 500 μL/min, Re=69.4). (**a**) Particle trajectory distributions at the trifurcated outlet; (**b**) grayscale value distribution across the channel width; (**c**) quantitative comparison of the purity and recovery rates for particles collected at the side and center outlets. Data are presented as mean ± SD (n=3 independent experiments), and the error bars represent the standard deviation.

## Data Availability

The original contributions presented in the study are included in the article. Further inquiries can be directed to the corresponding author.
